# PeSTo: parameter-free geometric deep learning for accurate prediction of protein binding interfaces

**DOI:** 10.1038/s41467-023-37701-8

**Published:** 2023-04-18

**Authors:** Lucien F. Krapp, Luciano A. Abriata, Fabio Cortés Rodriguez, Matteo Dal Peraro

**Affiliations:** Institute of Bioengineering, School of Life Sciences, Ecole Fédérale de Lausanne (EPFL) and Swiss Institute of Bioinformatics (SIB), Lausanne, 1015 Switzerland

**Keywords:** Molecular modelling, Proteins, Protein structure predictions

## Abstract

Proteins are essential molecular building blocks of life, responsible for most biological functions as a result of their specific molecular interactions. However, predicting their  binding  interfaces remains a challenge. In this study, we present a geometric transformer that acts directly on atomic coordinates labeled only with element names. The resulting model—the Protein Structure Transformer, PeSTo—surpasses the current state of the art in predicting protein-protein interfaces and can also predict and differentiate between interfaces involving nucleic acids, lipids, ions, and small molecules with high confidence. Its low computational cost enables processing high volumes of structural data, such as molecular dynamics ensembles allowing for the discovery of interfaces that remain otherwise inconspicuous in static experimentally solved structures. Moreover, the growing foldome provided by de novo structural predictions can be easily analyzed, providing new opportunities to uncover unexplored biology.

## Abstract (web summary)

Predicting protein interactions is crucial for understanding biological functions. Here, authors introduce a geometric transformer that accurately identifies protein binding interfaces, enabling new insights into unexplored biology.

## Introduction

Molecular interfaces are ubiquitous in biology and of utmost relevance beyond their central role in establishing cell boundaries and intracellular organization^1–3^. Especially so around proteins, which perform their functions by interacting with other proteins as well as with nucleic acids, membranes, and molecules and ions of various kinds.

Predicting the interactions that a given protein can establish with other molecules remains a major challenge in biology, still open despite numerous developments along various fronts^4–7^. The most modern methods for predicting protein interactions currently target the prediction of either specific pairs of interacting residues/atoms, relying intensively on the analysis of residue–residue coevolution patterns and thus limited to protein–protein interactions, or predicting only which regions of a protein are prone to interaction^7–14^. Even the latter, presumably a simpler problem, is yet far from solved, and most methods aim mainly at discovering protein interfaces tailored to interact with other proteins, with a strong focus on features of the protein surface and in some cases also exploiting their sequence conservation. These methods are thus limited, because calculation of protein surfaces and mapping of their properties are time-consuming, complicating their high-throughput application at the proteome scale; besides, they require parametrizations and are very sensitive to details and errors of the 3D structure or model^7,10,12–15^. Meanwhile, methods that rely on sequence conservation or residue coevolution often perform poorly for shallow sequence alignments. Approaches based on folding protein complexes de novo simultaneously discovering the interaction interfaces and subunit conformations, such as AlphaFold-multimer^16^, are limited to protein–protein interactions, are far slower than predicting the interaction interface from structures and will fail if the folding protocol itself fails.

Here, building on the recent successful application of transformers^17–20^ to various problems in natural language processing and protein structure prediction, we developed a rotation-equivariant transformer-based neural network that acts directly on protein atoms predicting interaction interfaces with high confidence, without the need for parameterization of the system’s physics, running fast enough to process large structural datasets such as ensembles from molecular dynamics simulations or entire foldomes. We build on this transformer to develop PeSTo—the Protein Structure Transformer—a generalized predictor of protein  binding  interfaces.

Trained to predict protein–protein interaction interfaces, PeSTo outperforms the state of the art. Training to predict other kinds of  binding interfaces was straightforward as the method does not depend on any explicit parametrization of physicochemical features. Therefore, confident predictions of protein interactions with nucleic acids, lipids, ligands and ions are also easily produced. Given the computational performance of the method, we could provide it not only as standalone code but also implemented in a user-friendly webserver [https://pesto.epfl.ch/]. PeSTo runs fast enough to allow processing of large volumes of structural data, such as molecular dynamics trajectories, enabling the discovery of cryptic interacting interfaces^21,22^, and the continuously growing foldome provided by AlphaFold predictions, which allows us to perform a detailed analysis of the human interfaceome.

## Results

### The Protein Structure Transformer (PeSTo)

Many successful methods combine transformers^17,18^ and geometric deep learning^7^ representing structures as graphs or point clouds and integrate the requirement of the invariance or equivariance of the neural network^23–29^. The major breakthroughs come from the field of protein folding^30^, where AlphaFold^19^ integrates attention in the Evoformer blocks and the structure module and the third track of the RoseTTAFold^20^ model uses a SE(3)-Transformer^31^ to refine the atom coordinates during folding. Moreover, the recurrent geometric network^32^ (RGN2) leverages the Frenet-Serret formulas to represent the backbone of proteins, and the geometric vector perceptron^33^ (GVP) uses linear operations to compose vector features with gating^34^. Multiple other machine learning-based protein–protein interaction site prediction methods have been developed^7,35–37^.

**Fig. 1:**
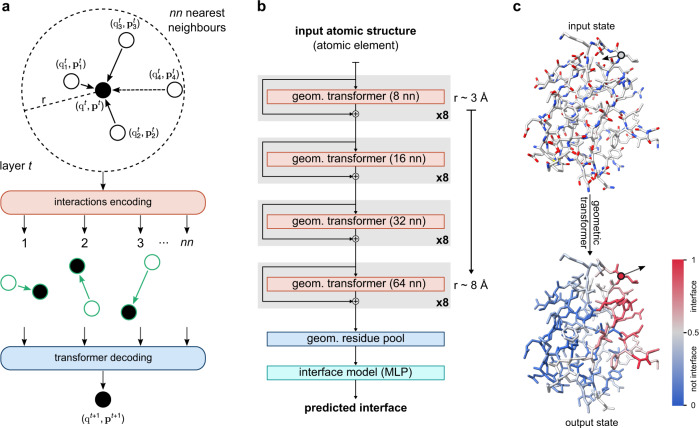
Overview of the PeSTo method. **a** Primary geometric transformer acting on the scalar and vectorial state of an atom at layer *t*. The interactions between the central atom and the nearest neighbors are encoded. A transformer is used to decode and filter the interactions information and to compute the new state of the central atom (Supplementary Algorithm 1). **b** The architecture of PeSTo for the prediction of interaction interfaces. The model is composed of multiple layers of geometric transformers with a set number of nearest neighbors (*nn*) and residual connections. The structure is reduced to a residue representation through an attention-based geometric pooling (Supplementary Algorithm 2). The residue states are collapsed, and the final prediction is computed from a multi-layer perceptron (MLP). **c** Example of application of the primary geometric transformer to all atoms in a structure.

We introduce here PeSTo, a parameter-free geometric transformer that acts directly on the atoms of a protein structure. As shown in Fig. 1 and detailed in Methods, the structure is represented as a cloud of points centered at the atomic positions, and its geometry is described through pairwise distances and relative displacement vectors which guarantee translation invariance. The atoms are described using only their elemental names and coordinates without any explicit numerical parametrization such as mass, radius, charge or hydrophobicity. Each atom is associated with a scalar state (*q*) and a vector state (*p*) encoding the properties of the structure. We define a geometric transformer operation acting on this cloud of points to update these states using the states and geometry in their local neighborhood as shown in Fig. 1a. The interactions between atoms for all nearest neighbors (*nn*) is encoded using the geometry (i.e., distance and displacement vector) and the state of the pair of atoms involved. A multi-head attention layer eventually decodes and regulates the propagation of the information (Supplementary Algorithm 1).

The geometric transformer operation is translation-invariant, rotation-equivariant and independent of the order of the atoms and order of the interactions. In order to retain the rotation equivariance of the vector states (see Supplementary Methods), the transformer attention linearly combines the scaled vectors from the local geometry and local state vectors to dynamically propagate vector state information based on the local context. The attention operation allows for a dynamic number of nearest neighbors (*nn*). However, in practice, the operation is much more computationally efficient with a fixed number of nearest neighbors. In the same fashion as applying convolution operations on an image, chaining geometric transformers can propagate information at a longer range than the local context of a single operation. Therefore, the main architecture is based on a bottom-up approach, starting from a small context of 8 nearest neighbors (≈3.4 Å radius) up to long-range interactions with 64 nearest neighbors (≈8.2 Å radius, Fig. 1b). The size of the context gradually increases allowing the model to progressively include more information while remaining cheaper in computation requirements and memory for deep models. The residual connection between geometric transformers enables to train deeper neural network architectures. Two additional modules aggregate the atom-based geometric description at the residue level independently of the number of atoms within a residue (i.e., geometric residue pooling, Supplementary Algorithm 2) and predict whether each amino acid is at an interacting interface or not (Fig. 1c).

In comparison with previous approaches like the SE(3)-transformer^31^ that uses spherical harmonics to encode geometrical context, our method simply uses vectors, modulating their information through the transformer attention. Compared to equivariant convolution, our method is based on graphs with geometry and performs message-passing using transformers.

### Protein–protein interface prediction

**Fig. 2:**
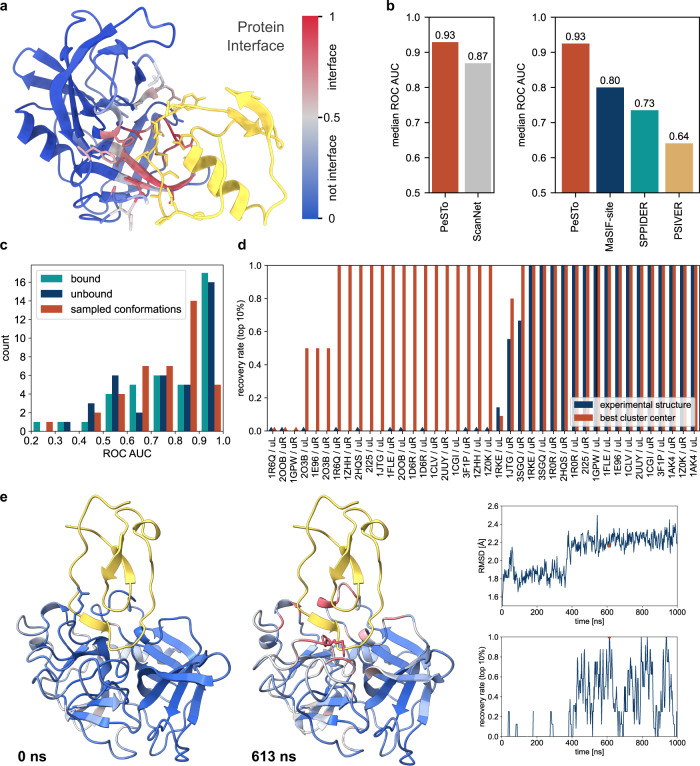
Assessment of protein–protein interface predictions with PeSTo. **a** Example of protein–protein interface prediction for the unbound conformation of Streptogrisin B (PDB: 2QA9) as can be retrieved at [https://pesto.epfl.ch/]. The confidence of the predictions is represented with a gradient of color from blue for non-interfaces to red for interfaces. The ligand in yellow was subsequently added based on the structure of the complex (PDB: 3SGQ) to show the quality of the prediction. **b** Comparison against other methods for protein–protein interface prediction. The methods are evaluated on PeSTo groundtruth on two different testing datasets for ScanNet and MaSIF-site. **c** Benchmark of our method on bound and unbound experimental structures, as well as their conformations sampled by 1 µs-long MD simulation for 20 complexes taken from the PPDB5. **d** Recovery rate (considering top 10% predicted residues) for the clustering of predicted interfaces on 1 µs-long MD simulations of the unbound state only, compared to the predicted interface of the experimental structure for the unbound receptor (uR) and ligand (uL) for 20 complexes taken from the PPDB5. **e** Protein–protein interface prediction on the experimentally resolved structure of unbound porcine pancreatic elastase (left, PDB: 9EST) and an open conformation sampled using MD (center) and selected using clustering on the conformations. The ligand in yellow was subsequently added based on the structure of the complex (PDB: 1FLE) to show the quality of the prediction. R217 is shown in licorice to illustrate the rearrangement of the loop region. (Right) The root mean square deviation (RMSD) from the experimental unbound conformation and recovery rate average over 4 frames are shown as a function of the simulation time (the red dots indicate the selected snapshot shown in (**d**)). Source data are provided as a Source Data file.

We trained a PeSTo model using over 300’000 protein chains from the PDB (see “Methods”) to predict which residues are involved in a protein–protein interface as flagged by an output value ranging from 0 to 1 (Fig. 2a). Zero means that the residue is predicted to not be engaged in interactions, while values of 1 predict the residue to be at an interface. In practice, the actual value of the prediction reflects the confidence of the prediction at the residue level, such that values farther away from 0.5 imply higher confidence, see Supplementary Fig. 1.

We first evaluated the performance of PeSTo against the most recent method develop to address a similar task, namely ScanNet^15^. We used a benchmark dataset of 417 structures commonly shared by the two methods (see “Methods”). On this benchmark PeSTo outperforms ScanNet without multiple sequence alignment (MSA) with a median receiving operating characteristic (ROC) area under the curve (AUC) of 0.93 against 0.87 (Fig. 2b and Supplementary Table 1 for an extended comparison on different datasets and metrics including precision-recall AUC and Matthews correlation coefficient). Moreover, we compared the speed of the two methods quantitatively (Supplementary Fig. 2), finding that the average runtime for PeSTo (5.3 ± 2.8 s) and ScanNet without MSA (9.1 ± 1.8 s) on CPU are comparable. However, the runtime of ScanNet with MSA (160 ± 83 s) is two orders of magnitude slower than PeSTo, providing no substantial improvement against PeSTo according to all metrics (Supplementary Table 1).

We further compared PeSTo on the same dataset used to benchmark MaSIF-site^7,36^ (one of the best algorithms currently available), which we excluded from our training set at 30% sequence identity. PeSTo reaches a median receiving operating characteristic (ROC) area under the curve (AUC) of 0.92 against 0.8 for MaSIF-site followed by SPPIDER^35^ and PSIVER^37^ (Fig. 2b). The interfaces predicted by PeSTo have a higher ROC AUC than all other methods benchmarked here for 38 out of 53 structures.

Finally, we compared the protein–protein interfaces as predicted by PeSTo against those predicted by AlphaFold-multimer. We selected 23 dimers (i.e., 46 interfaces) from the structures within the validation set of PeSTo and AlphaFold (see “Methods”). We observed that PeSTo performs almost as well as AlphaFold-multimer (see Supplementary Table 2) without the additional cost of computing any multiple sequence alignment. These results show therefore how our method can be used to quickly screen for potential interfaces with an accuracy comparable to AlphaFold-multimer.

To further showcase the quality of the predictions in real-world applications, we tested proteins from the Protein-Protein Docking Benchmark 5.0^38^ (PPDB5) dataset in their unbound conformations. The example in Fig. 2a shows PeSTo recovering the interaction interface of Streptogrisin B with ovomucoid from its unbound conformation (0.93 Å RMSD from the bound state) with a ROC AUC of 0.96. Overall, on the whole PPDB5 dataset composed by a variety of targets of variable difficulty for the general task of protein–protein docking, PeSTo reaches a median ROC AUC of 0.78 for predictions on the unbound structures and 0.85 for the respective bound states.

Importantly, the short time needed to run the model (i.e., 300 ms for a 100 kDa protein from PDB load to prediction on a single NVIDIA V100 GPU, Supplementary Fig. 3) allows us to evaluate snapshots from large structural ensembles efficiently, extracted from molecular dynamics (MD) simulations. We applied PeSTo for protein–protein interface prediction on conformations sampled by 1 µs-long atomistic MD simulations of the experimentally derived unbound and bound subunits of 20 selected binary complexes taken from the PPDB5 (Fig. 2c). The bound and unbound structures along with the MD-sampled conformations reach a median ROC AUC of 0.85, 0.82 and 0.79, respectively (see Supplementary Table 3 for additional metrics). We observe that the model performs almost as well on experimentally solved bound and unbound conformations. Although overall the ROC AUC decreases with a higher RMSD from the bound structure (Supplementary Fig. 4), our method is still able to recover the interface with a ROC AUC higher than 80% for most structures and MD-sampled conformations.

In some cases, processing MD trajectories of unbound proteins with PeSTo identifies certain interfaces better than when PeSTo is run on the starting static structure, which suggests an impactful practical application of our method to real-life situations (Fig. 2d). Striving to provide a protocol for every-day applications of PeSTo, we consider that a user might look for a handful of high-ranked residue predictions to characterize the binding interface. We define therefore the “recovery rate” as the ability to predict the 10% high-ranked residues, which in the case of our MD dataset correspond to 3 ± 2 residues. If all these residues are predicted correctly, we consider that the interface is fully recovered. Out of 20 complexes composed by 40 constituent subunits and relative interfaces, the model has a perfect recovery rate for 16 interfaces when applied straightaway on the experimental structures of the unbound subunits. Out of the remaining 24 cases, we show that it is possible to fully recover the binding interface for additional 16 subunits (80%) using MD to more extensively sample the protein conformation landscape and clustering to further group predicted interfaces.

For instance, PeSTo predicts no interface for the experimentally solved structure of the unbound porcine pancreatic elastase (PDB ID 9EST) (Fig. 2e). The unbound experimental conformation has a backbone RMSD of 1.2 Å from the bound complex with elafin (PDB ID 1FLE). However, MD simulation starting from the unbound porcine pancreatic elastase alone shows a conformational switch leading to the recovery of the interaction interface with elafin with a cluster center ROC AUC of 0.92 and perfect recovery rate of predicted binding interface (i.e., 3 residues in this case). Inspecting the MD simulation unveils that the motion of a loop in elastase is required to allow elafin to enter the pocket and accommodate an inter-molecular β-sheet that stabilizes the complex as solved experimentally.

### General protein binding interface prediction

**Fig. 3:**
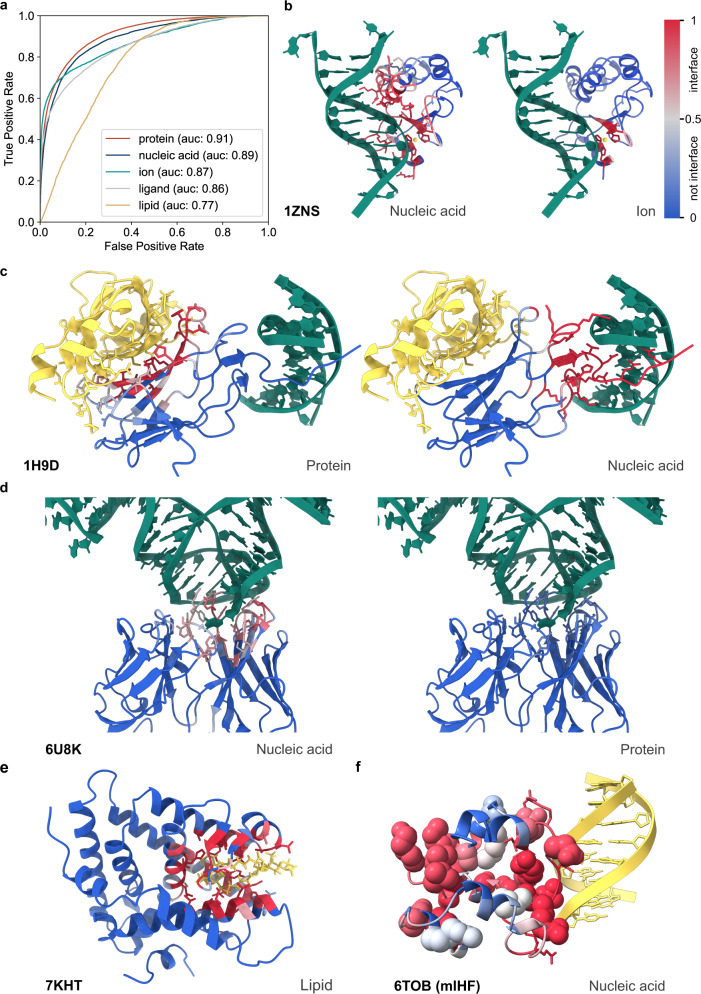
General protein binding interface prediction with PeSTo. **a** ROC curve for the predictions of different types of interfaces with PeSTo. (**b**–**f**) Examples of predicted binding interfaces. The confidence of the predictions is represented with a gradient of color from blue for non-interfaces to red for interfaces. The structures in yellow and green were added subsequently from the reference complexes. **b** Colicin E7 endonuclease domain in complex with DNA and a zinc ion (PDB: 1ZNS). **c** core-biding factor subunit alpha-2 in complex with core-binding factor subunit beta and DNA (PDB: 1H9D). **d** Antigen-binding fragment in complex with RNA (PDB: 6U8K). (**e**) Steroidogenic factor 1 bound to a phosphoinositide (PDB: 7KHT). **f** Predicted interface with nucleic acid for the mycobacterial integration host factor (: 6TOB). Residues observed to bind DNA through solution-state NMR are represented with spheres. The DNA molecule is modeled from an X-ray structure of the protein homolog from *S. coelicolor*, crystallized with DNA (PDB: 4ITQ). Source data are provided as a Source Data file.

In light of the results for protein–protein interface predictions, we extended the model to find and identify more types of interfaces, resulting in a generalized PeSTo model that predicts protein interaction interfaces with other proteins as well as with nucleic acids, ions, ligands, and lipids. We trained a generalized PeSTo model with PDB structures featuring all the kinds of expected interactions, as described in Methods. The interface predictions for protein–nucleic acid interfaces are almost as good as for protein–protein interfaces, reaching ROC AUC of 0.89 for the testing set (Fig. 3a). The generalized model can also detect ion, ligand, and lipid interfaces with ROC AUCs of 0.87, 0.86, and 0.77, respectively on each testing set (see Supplementary Table 4 for additional metrics). The model does experience some confusion between ions and ligands as revealed by the confusion matrix (Supplementary Fig. 5). Poorer performance on protein–lipid prediction depends on the quite limited number of protein–lipid complexes available so far in the PDB (only 0.7% of the utilizable data we compiled). We note that retraining the model on the same dataset but with a maximum of 5% sequence identity instead of 30% between training, validation and test sets results in equivalent performances within ±1% ROC AUC in average over all interfaces prediction type, confirming PeSTo stability over homology reduction.

We next illustrate the generalized PeSTo model showcasing five examples from the testing set that attest to its capacity to discern among various interfaces, even when they are overlapping or under-represented in the PDB. The first example (Fig. 3b) corresponds to the colicin E7 endonuclease domain, which binds DNA through an interface that includes a zinc ion (PDB ID 1ZNS). Running the apo-protein through the generalized PeSTo returns correct predictions for both interfaces, even in the overlapping part. The second case (Fig. 3c) corresponds to the complex formed by RUNX1 with a dsDNA bound to one end and the protein CBFβ bound to the other (PDB ID 1H9D). Running the isolated RUNX1 through the generalized model returns clear, accurate interfaces through the DNA and protein channels. In the third example (Fig. 3d) we challenge the generalized model with the structure of an antibody that binds RNA (PDB ID 6U8K) as opposed to most available antibodies which are bound to other protein targets. The generalized model correctly predicts no interface for proteins and the correct interface for RNA.

Although on interfaces with lipids the generalized PeSTo performs less well, in practice we observe that the model is able to accurately detect lipid-binding pockets for soluble proteins (exemplified by the steroidogenic factor in Fig. 3e) and even the membrane-spanning regions of transmembrane proteins (Supplementary Fig. 6). Despite not specifically trained for any of these, in both cases PeSTo is able to detect specific pockets for lipids with stronger scores. We note that many protein interfaces with lipids are only partially evident in PDB structures (for example a single lipid bound to a membrane-spanning region), resulting in low training data quality thus leading to an artificial drop of the ROC AUC.

Interestingly, we also find that PeSTo extends its prediction power over its own training, as exemplified for the case of a DNA-binding bacterial integration host factor (mIHF) for which an X-ray structure of the DNA-bound form was available (Fig. 3f). This structure presents in the biological assembly one DNA-binding interface^39^ that was included in the training set, but solution-state NMR titrations have shown a far more extensive interaction surface, mainly spread over two surface patches as required to bend DNA as demonstrated by AFM^40^. PeSTo’s predictions for this protein go beyond its training, pointing at two surface patches that match very well with the NMR data in solution.

### High-throughput prediction of binding interfaces for the human proteome

We sought to explore the whole human proteome and analyze what we call hereafter the interfaceome, namely all the potential protein interfaces able to bind other proteins, nucleic acids, lipids, ligands and ions. For this task, we obtained all the structures and models for human proteins in the AlphaFold-European Bioinformatics Institute (AF-EBI) database^19,41^. The database currently includes highly accurate structures, many actually containing domains with experimentally solved structures, models with no structures in the PDB or with little homology to PDB structures yet highly accurate as judged by AlphaFold predicted local distance difference test (pLDDT) and predicted alignment error (PAE), and also several models of very low pLDDT and PAE scores. We selected 7464 high-quality models for further analysis from the total of 20504 entries based on their pLDDT and PAE scores, as described in Methods.

**Fig. 4:**
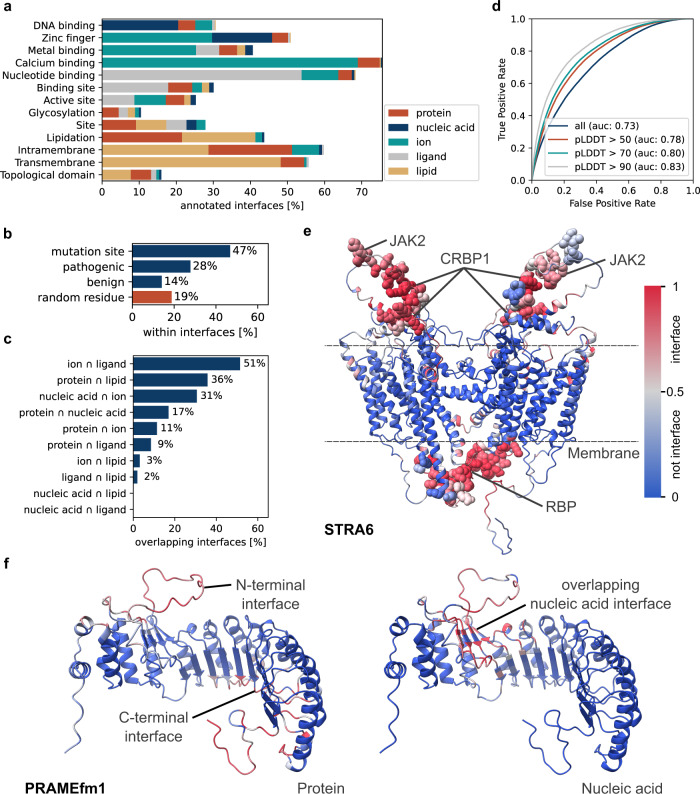
PeSTo-based analysis of the human proteome. **a** Percentage of entries with specific UniProt features for which PeSTo predicts an interaction interface at the annotated sequence region. **b** Percentage of sites with mutations, pathogenic or benign natural variants within a predicted interface. The baseline is the probability of a random residue being within an interface. **c** Percentage of overlapping interfaces for all 10 pairs of five interface types. **d** Comparison of predicted protein-binding interfaces from PeSTo using the models of yeast protein complexes predicted by Humphreys et al.^42^. Regions of the predicted structures are filtered out at different pLDDT thresholds. **e** Human receptor for retinol uptake (STRA6, UniProt Q9BX79). Protein interfaces predicted with PeSTo. Sites of interest as described by Berry et al.^43^ are highlighted with spheres and are consistently found by PeSTo predictions. See also Supplementary Fig. 23. **f** PRAME family member 1 (PRAMEfm1, UniProt O95521) predictions for protein (left) and nucleic acid (right) interfaces. See also Supplementary Fig. 24. Source data are provided as a Source Data file.

We could immediately notice that our model produces robust results that further validate the quality of interface predictions. In particular, the amino acid distributions for specific molecular interfaces recapitulates known biochemistry (e.g., Arg and Lys residues are mostly engaged in nucleic acid interactions, hydrophobic amino acids in lipid-binding sites, etc., see Supplementary Figs. 7, 8). Furthermore, mapping the predicted interfaces to UniProt-annotated features showed strong agreement with the expected functional roles of the binding interfaces (Fig. 4a and Supplementary Data 1). Additional support for the quality of the predictions came from the mapping of the predicted interfaces and their subcellular localizations, GO functions and processes (Supplementary Figs. 9–19).

We interrogated further the human interfaceome for the geometrical features of the predicted interfaces and observed that when computing their solvent-accessible surface areas (SASA), interactions with proteins and nucleic acids involve the largest areas with 32 ± 22 and 29 ± 23 nm^2^, respectively, while ligands and ions involve small pockets of 16 ± 7 and 7 ± 4 nm^2^. The SASA distribution for protein–lipid interactions has instead a bimodal distribution that reflects specific lipid-binding sites (17 ± 9 nm^2^) and large lipid coronas surrounding transmembrane protein domains (75 ± 19 nm^2^, Supplementary Fig. 20).

As further validation, extending the analysis to another eukaryotic proteome, we compared PeSTo predictions to the available predictions of protein binary complexes of the yeast proteome derived with AlphaFold and RoseTTAFold^42^. Also in this case, we observed a very good correlation between sets of residues involved in interfaces with the ROC AUC steadily increasing as the analysis is limited to regions of the models of higher quality (Fig. 4d). Moreover, we identified additional binding interfaces that can extend further the interaction network of binary complexes and can be used as complementary means to better describe and model the architecture of large protein complexes (Supplementary Fig. 21).

Notably, 47% of the UniProt annotations for mutation sites fall in a predicted interface, 28% correspond to pathogenic natural variant sites, and 14% to benign natural variant sites with a baseline of 19% for random residues being within an interface (Fig. 4b). As we make all these predictions fully available in the PeSTo website and the underlying structural models are freely available in the EBI database, it is straightforward for cell biologists to consult where exactly these pathogenic mutations fall and what interactions they might compromise, in order to develop rational working hypotheses that could help further therapeutic development.

Carrying on to a large-scale analysis of the predicted interfaces, we observed strong segregation for certain kinds of interfaces and a quite large overlap for others (Fig. 4c and Supplementary Fig. 22). An example of the former case is that of protein interfaces prone to interact with proteins or with ions/ligands, which are highly segregated. Studying these patterns further could potentially help in the discovery of allosteric regulation mechanisms. Among pairs of interfaces that feature a quite extensive overlap are those that mediate interactions with other proteins and with lipids, which could possibly point at reversible protein dimerization/oligomerization at membranes. On actual application of PeSTo to address biological questions, specific cases shall be looked at carefully, and overlaps or lack thereof might bring information as exemplified next.

Importantly, the availability of high-resolution structures and high-quality AlphaFold models of the human proteome, as well as other proteomes, provides biologists with the opportunity to immediately and easily interrogate specific interaction predictions of their proteins of interest, developing quickly working hypotheses, and designing new experiments, allowing in turn to discover new biology. Among multiple interesting examples, we highlight here two cases of proteins that lack structures in the PDB but where the application of PeSTo to AlphaFold models proposes clear prompts to drive forward biological studies: the human receptor for retinol uptake STRA6 (UniProt Q9BX79, Fig. 4e) and the PRAME family member 1 (PRAMEfm1, UniProt O95521, Fig. 4f).

STRA6 is modeled in the AlphaFold—EBI database as a monomer, although one would expect it to be most likely a dimer like most small-molecule transmembrane transporters. We applied PeSTo to the model as provided (i.e., as monomer) and to a model of the dimer built with AlphaFold-multimer. PeSTo predicts in both cases interfaces prone to interact with other proteins and with lipids. In the monomeric model, part of the interface predicted to interact with lipids overlaps with an interface predicted for proteins, suggesting this is the region for homodimerization within the membrane. Accordingly, this interface is not predicted for the dimer, and the new set of residues predicted to interact with lipids makes perfect sense as the membrane-spanning region (Supplementary Fig. 23). Another set of residues predicted to form interfaces for protein binding map to 4 locations outside the transmembrane region (Fig. 4e). On the cytoplasmic side of the membrane, three STRA6 segments with strong predicted potential for protein interaction map to a site made up of two folded elements that overlap with sequence segments that Berry et al.^43^ actually proposed as a binding site for regulator cellular retinol-binding protein 1 (CRBP1), next to a predicted interaction site that corresponds to a known kinase binding site (JAK2). On the extracellular side of the membrane, a binding site expected for the carrier retinol-binding protein (RBP) is also predicted. Therefore, residues with high protein interaction scores (e.g., K324-K348 for the reported RBP, L251-R257 and R638-L46 around the reported CRBP1 site, and D612-K626 for the kinase site, Fig. 4e) are potential candidates for mutagenesis studies aimed at probing the various interactions.

The second example worth describing, PRAMEfm1, is annotated in UniProt as presumably linked to cell differentiation, proliferation, and apoptosis processes through negative regulation of transcription. The protein has very weak sequence homology to some ribonuclease inhibitors, and a high-confidence AlphaFold model finds substantial structural similarity to some of them except for certain insertions and deformations in the N-terminal half. Contrary to PeSTo’s predictions on the ribonuclease inhibitor, which are perfectly confined to the known ribonuclease binding interfaces (Supplementary Fig. 24), on AlphaFold-EBI’s model for PRAMEfm1 PeSTo detects two clear regions prone to protein interactions. A region in the C-terminal half could accommodate proteins similar to how the ribonuclease inhibitors interact with ribonucleases, immediately suggesting a set of residues whose mutations would disrupt the interaction with a protein target on this side (e.g., rim made of short stretches of β-turns around H243, T278, G303, Q360, N387, L422, T455, and the C-terminal P464-L472). The second protein interface predicted by PeSTo maps to the N-terminal half, it includes a short segment of low confidence, possibly disordered, and overlaps with a large surface region predicted by PeSTo to bind nucleic acids. This stands as another clear region whose roles could be explored experimentally by targeting residues L122-Q145 and possibly the connecting β-strands too. Although it is hard to advance specific roles for PRAMEfm1 from this computational analysis, in the context of the UniProt annotations PeSTo predictions would suggest a role as a hub connecting other proteins (through the C-terminal half) to nucleic acids (through the N-terminal half), likely RNA given its cytoplasmic localization, and possibly regulated by other proteins that also bind to the N-terminal half.

We finally compared protein–protein interface predictions of PeSTo with modeling protein–protein interactions using AlphaFold-multimer^16^, a procedure richer in information as including also evolutionary couplings. On the STRA6 example, AlphaFold-multimer predicts binding of CRBP1 onto STRA6 around the same residues that we discuss from literature, i.e. essentially the same prediction as PeSTo. However, AlphaFold-multimer does not predict any interaction at all for JAK2 and predicts an incorrect binding site for RBP. In the case of PRAMEfm1, we detect a plausible interface for nucleic acid binding, which AlphaFold is not trained to predict, and we detect a protein interaction region of high confidence but without any information about the identity of the partners, precluding to test with AlphaFold any obvious, specific complex. These comparisons highlight a synergic intersection between PeSTo and AlphaFold-multimer for the prediction of protein–protein interactions. Namely, PeSTo can produce predictions that are consistent with the reported biochemistry, while AlphaFold-multimer can interrogate these binding interfaces when the network of interactions is known.

## Discussion

We showed here that a geometrical transformation of protein atomic coordinates suffices to detect and classify protein binding interfaces at high resolution, surpassing the prediction capabilities of other methods without the need of explicitly describing the physics and chemistry of the system, hence without the overhead of pre-computing molecular surfaces and/or additional properties. All this with modest computational resources and at a very high speed that enables the analysis of large structural ensembles, for example those produced by molecular dynamics simulations, which discloses the opportunity to investigate the dynamic features of protein interaction networks. Likewise, large structural datasets, like those being created by the latest generations of tertiary protein structure prediction tools, can be easily analyzed, as done here for the human foldome, with the possibility to quickly access new biological discoveries.

To make PeSTo-based predictions for proteins available to the community, we implemented it in a webserver at https://pesto.epfl.ch/, accessible free of charge without registration. The server takes any protein structure and model in PDB format (uploaded or fetched from the PDB or the AlphaFold-EBI databases) and returns them with additional information reporting on the confidence of the prediction on a per-residue basis. Output files can be downloaded or visualized right within the website. Furthermore, we provide the source code (https://github.com/LBM-EPFL/PeSTo) as to facilitate application to large structural ensembles as done here for the human interfaceome.

Provided that sufficient training data are available, the method can be easily upgraded (as for instance to improve further protein–lipid predictions) and is reusable for other specific applications. In fact, the parameter-free PeSTo architecture is general enough that could be easily accommodated to pursue other structure-based problems such as docking or modeling interactions with materials. The description is totally agnostic to the exact physicochemical properties of the atoms in the structure, thus easily extendable to other materials and fields, and is probably also less sensitive to problems related to the starting structures such as missing atoms as compared to methods that require intermediate calculations of surfaces and volumes.

Given the ever-growing accumulation of structural information and rapid expansion of predicted foldome data, PeSTo stands as an accurate, flexible, fast, and user-friendly solution to dissect the vast and dynamic interaction landscape of proteins and can be readily used to discover new and richer biological insights.

## Methods

### Datasets

The dataset is composed of all the biological assemblies from the Protein Data Bank^44^. The subunits are clustered using a maximum of 30% sequence identity between clusters. The clusters of subunits are grouped into approximately 70% training set (376216 chains), 15% validation set (101700 chains), and 15% testing set (97424 chains). We selected the best hyperparameters by evaluating the model on the validation set. The testing set is composed of the clusters containing any of the 53 subunits from the MaSIF-site benchmark dataset or 230 structures from the Protein-Protein Docking Benchmark 5.0^38^ (PPDB5) dataset. Additionally, we extracted a subset 417 structures common in the benchmark dataset of ScanNet^15^ and the testing dataset of PeSTo. Unless specified, all the examples selected to assess the quality of the predictions from the model belong to the testing set.

### Structure processing

All models of the structures are loaded as a single structure. The chain name is tagged with the model identifier to distinguish subunits from different models. Moreover, the chain name of all non-polymer chemical molecules is tagged to have them in separate subunits. Duplicated subunits, molecules, and ions generated when concatenating multiple models are removed. The first alternate location of the atoms is kept. Water, heavy water, hydrogen, and deuterium atoms are removed from the structures.

### Features and labels

We identified the 30 most common atomic elements on PDB. The element is used as the only feature as a one-hot encoding. The input vectorial features are initially set to zero. The distances matrices and normalized displacement vector matrices are used as geometrical features. Amino acids, nucleic acid, ions, ligands, and lipids are selected from a list of 20, 8, 16, 31, and 4 most common molecules, respectively. Non-native molecules used to help solving the structure are ignored. An interface is defined as a residue–residue contact within 5 Å. All protein–protein interfaces as well as protein–nucleic acid, protein–ion, protein–ligand, and protein–lipids interfaces are identified. The details of the interface for each subunit are stored in the dataset as an interactions types matrix (79×79). This enables the selection of specific interfaces as labels at the start of the training session without having to rebuild the whole dataset. The interfaces targets can be selected from any combinations of subsets from the 79 molecules available.

### Protein Structure Transformer architecture

The input features are embedded to an input state size of *S* = 32 with a 3 layers neural network with hidden layer size of 32. Each geometric transformer is composed of 5 neural networks of 3 layers to perform the multi-head self-attention (*S* = 32, *N*_key_ = 3, *N*_head_ = 2) as described in Supplementary Algorithm 1. For structures with a number of atoms smaller than the set number of nearest neighbors (*nn*), the additional non-existent interactions are sent to a sink node with a scalar and vector state set to zero. 4 sets of 8 geometric transformers with an increasing number of nearest neighbors for each set (*nn* = 8, 16, 32 and 64) are applied in succession. The geometric residue pooling module aggregates the encoding at the atomic-level of the structure to a residue-level description by using a local multi-head mask on the atoms forming each residues (*S* = 32, *N*_head_ = 4) as described in Supplementary Algorithm 2. The last module is a multi-layer perceptron with 3 layers of hidden size of *S* = 32 decoding the state of all residues and computing the prediction, returning a confidence score from 0 to 1.

### Training

The model is trained to predict protein interfaces with protein, nucleic acid, ligand, ion, or lipid. The best neural networks architecture was trained for 8 days on a single NVIDIA V100 (32 GB) GPU. Subunits with a maximum of 8192 atoms (≈100 kDa) without hydrogens are used to limit the memory requirement during training. Subunits with less than 48 amino acids are ignored during training. We trained only on the first bioassembly provided by the PDB database. The effective generalized protein interfaces dataset after filtering is composed of 113805 subunits for training and 29786 subunits for testing.

### Evaluation

Our method was compared with ScanNet^15^, MaSIF-site^7,36^, SPPIDER^35^ and PSIVER^37^. ScanNet is the most recent geometry-based deep learning method for protein–protein interface prediction. MaSIF-site is the best available surface-based deep learning method for protein–protein interface prediction. SPPIDER is a long-standing and well-tested method used as a reference for protein–protein interface prediction. PSIVER only uses sequence information and is benchmarked to show the difference in performance between structure-based and sequence-based methods. The benchmarking of PeSTo was performed using structures taken from the testing dataset exclusively. We use 512 structures per interface type for the protein, ion and ligand interfaces predictions. The low amount of structures available limits the testing dataset to 391 and 161 structures for the nucleic acid and lipid interfaces prediction, respectively.

### AlphaFold-multimer benchmark

We identified 23 dimers (i.e., 46 interfaces) not present in the training set of PeSTo or of AlphaFold and with a maximum of 20% sequence identity with the AlphaFold-multimer training set (i.e., structures published up to April 30th 2018)^16^. We modeled the protein complexes using the implementation of AlphaFold-multimer by ColabFold with MMseqs2^45^ with the default parameters of 10 recycles and 5 predicted models. We extracted the protein–protein interfaces of the AlphaFold-multimer models (i.e., residue–residue contacts within 5 Å) and computed the average interfaces over the 5 predicted models. PeSTo was used to predict the protein–protein interfaces for the 46 subunits. Lastly, we computed the accuracy, precision, Matthews correlation coefficient (MCC), receiver operating characteristic (ROC) and precision-recall (PR) area under the curve (AUC) on the PeSTo predicted protein–protein interfaces and the average protein–protein interfaces of the AlphaFold-multimer predicted models, which are reported in Supplementary Table 2.

### Molecular dynamics simulations

20 complexes from the PPDB5 dataset were selected based on the resolution of the structure and the difficulty to parametrize. For each, we performed a classical 1 µs-long MD simulation in the NpT ensemble (at 1 atm and 300 K, after NVT equilibration over 2 ns and with settings as in ref. ^46^) of the subunits alone for the bound receptor (bR), unbound receptor (uR), bound ligand (bL), and unbound ligand (uL). All systems were setup using CHARMM36m^47^ and its recommended TIP3P water model, and MD simulations were run with Gromacs 2020^48^. For the general analysis, 500 frames per simulation are used to evaluate PeSTo for a total of 400,000 frames (Fig. 2c), which are further clustered using the CLoNe algorithm^49^ for the analysis of the unbound states (Fig. 2d).

### Human interfaceome

We downloaded all the available 20’504 (at the time of writing) AlphaFold predicted structures version 2 for human sequences from the AlphaFold-European Bioinformatics Institute (AF-EBI) database^19,41^. The same pipeline and data analysis can be applied to any organism. The most accurate AlphaFold structure models are selected with a minimum of 70% of the structure with a pLDDT > 70 and average PAE < 10 Å in the well-folded regions (pLDDT > 70). The analyzed dataset is composed of 7464 quality predicted structures from a total of 20,504.

PeSTo was applied to all models. For the analysis of interface residue composition and UniProt-annotated sequence regions, we considered only predicted interfaces with high confidence (>0.8) at well-folded regions (pLDDT > 70). Interface residues are grouped into interfaces by connecting all residues at well-folded regions (pLDDT > 70) and at a predicted interface (>0.5) with alpha carbon within 10 Å. We selected only the predicted interfaces of quality with average predicted interface confidence above 0.8 for all analyses. Two quality interfaces of different types are overlapping if they share at least 5 residues. Solvent-accessible surface area per atom of all models was computed using the Shrake and Rupley algorithm^50^ implemented by MDTraj^51^.

The UniProt-annotated features and GO terms for all corresponding 20’504 AlphaFold models were downloaded from UniProt website^52^. The features analyzed include a curated list of annotated features, the subcellular localization, the mutation sites, natural variants, and the GO biological process and molecular function. The pathogenicity of natural variants was extracted from the clinical significance of genetic variations available at dbSNP^53^.

We downloaded all the 1102 predicted yeast protein complexes with AlphaFold and RoseTTAFold by Humphreys et al.^42^, and extracted the interfaces from the predicted complexes with an interface defined as a residue–residue contact within 5 Å. PeSTo was applied to predict the protein–protein interfaces on the subunits of the predicted complexes alone.

### Reporting summary

Further information on research design is available in the Nature Portfolio Reporting Summary linked to this article.

## Supplementary information

Supplementary Information

Peer Review File

Description of Additional Supplementary Files

Supplementary Data 1

Reporting Summary

## Data Availability

We used freely available data as described in Methods. The data and code to reproduce the datasets and experiments are available at [https://github.com/LBM-EPFL/PeSTo]. A previously published structure can be accessed via the accession codes 2QA9, 3SGQ, 1FLE, 9EST, 1ZNS, 1H9D, 6U8K, 7KHT, 6TOB, and 4ITQ. Source data are provided with this paper.

## Source data

Source Data

## Code availability

The source code is available [https://github.com/LBM-EPFL/PeSTo] and implemented in the webserver [https://pesto.epfl.ch/]. An archived version of the code used to produce the results presented in this work is available at [10.5281/zenodo.7728869].
