# Supplementary material for: PeSTo: parameter-free geometric deep learning for accurate prediction of protein binding interfaces
Source: Nat Commun. 2023 Apr 18;14:2175. doi: 10.1038/s41467-023-37701-8 (PMC10113261; doi:10.1038/s41467-023-37701-8)
Supplement: Supplementary file 1 — Supplementary Information [file 41467_2023_37701_MOESM1_ESM.pdf]

PeSTo: parameter-free geometric deep learning for accurate prediction of protein binding interfaces

Lucien F. Krapp, Luciano A. Abriata, Fabio Cortés Rodriguez, Matteo Dal Peraro*

Institute of Bioengineering, School of Life Sciences, Ecole Fédérale de Lausanne (EPFL) and Swiss Institute of Bioinformatics (SIB), Lausanne 1015, Switzerland

* To whom correspondence should be addressed: M.D.P., email: matteo.dalperaro@epfl.ch

Supplementary information

- Supplementary Methods;
- Supplementary Algorithm 1-2;
- Supplementary Tables 1-4;
- Supplementary Figures 1-24;
- Supplementary Material attached (i.e., curated table of all human protein interfaces predicted by PeSTo).

Supplementary Methods

Translation invariance and rotation equivariance of PeSTo

1. **Translation invariance.** The translation invariance is directly provided by the geometrical features used. The neighbors distances (d_{nn}) and normalized relative displacements ($\boldsymbol{r}_{nn}$) are independent of the origin of the coordinate system. The distance is defined as $\| x_i - x_j \|$. The normalized relative displacement is defined as $r_{ij} = (x_i - x_j) / \| x_i - x_j \|$.

2. **Rotation equivariance.** In order to guarantee the rotation equivariance, all operations on vectors in PeSTo are a combination of three rotation invariant and equivariant operations: namely, the scalar product (invariant), the scalar multiplication (equivariant), and the linear combination of vectors (equivariant). It follows that the norm ($\|\cdot\|$), the element-wise product ($\odot$) and the projection from N to M vectors (i.e., $P\boldsymbol{X}$ with $X \in \mathrm{R}^{N\times3}$ a vector and $P \in \mathrm{R}^{M\times N}$ a projection) are also rotation invariant, equivariant and equivariant, respectively. Therefore:

2.1 **Rotation equivariance in geometric transformer** (see **Supplementary Algorithm 1**).

- The packed nodes and edges features (X_n, X_e) are composed of rotation invariant features (d_{nn}, q, q_{nn}) and rotation invariant quantities derived from scalar product of ($\boldsymbol{r}_{nn}$, $\boldsymbol{p}$, $\boldsymbol{p}_{nn}$).
- The encoded geometric feature ($\boldsymbol{X}_g$) is a vector quantity composed of the input vector states ($\boldsymbol{p}$, $\boldsymbol{p}_{nn}$) and element-wise scalar multiplication of the normalized relative displacement vectors ($\boldsymbol{r}_{nn}$).
- The vectorial hidden state ($\boldsymbol{p}_h$) is obtained by two equivariant projections. First the attention is a projection from the *3n* neighborhood vectorial features to N_h attention heads (equivariant). Then the W_{ppm} projects the states (S) for all heads (N_h) channels into the final output state of size S (equivariant).
- The final output for the vector state (**p**') is obtained by linear combination of the previous state (**p**) and the vectorial hidden state ($\mathbf{p}_h$)

2.2 **Rotation equivariance in geometric pooling** (see **Supplementary Algorithm 2**).

- The packed node features (X_n) is composed of rotation invariant features (q) and rotation invariant quantities derived from scalar product of ($\boldsymbol{p}$).
- The final output for the vector state at the residue level ($\boldsymbol{p}_r$) is obtained by two equivariant projections. First the attention is a projection from the N atoms to N_r residues vectorial features for N_h attention heads (equivariant). Then the W_{prpm} projects the states (S) for all heads (N_h) channels into the final output state of size S (equivariant).

2.3 **Rotation invariance in the interface model.**

- The input features of the multilayer perceptron of the interface model are the scalar state at the residue level (q_r) and the norm of the vector state at the residue level ($\boldsymbol{p}_r$) both rotation invariant.

Algorithm 1: Geometric transformer

Input: Center node features: $q \in \mathbb{R}^{N\times S}, p \in \mathbb{R}^{N\times S\times 3}$
Context neighbors features: $q_{nn} \in \mathbb{R}^{N\times n\times S}, p_{nn} \in \mathbb{R}^{N\times n\times S\times 3}$
Geometry features: $d_{nn} \in \mathbb{R}^{N\times n}, r_{nn} \in \mathbb{R}^{N\times n\times 3}$
Output: New state of center node: $q', \vec{p}'$

`// pack node and edges features`
$X_n \leftarrow concat(q, \|\vec{p}\|) \in \mathbb{R}^{N\times 2S}$ ▷ Node features
$X_e \leftarrow concat(d_{nn}, q, \|\vec{p}\|, q_{nn}, \|\vec{p}_{nn}\|, \vec{p}\cdot\vec{r}_{nn}, \vec{p}_{nn}\cdot\vec{r}_{nn}) \in \mathbb{R}^{N\times n\times 6S+1}$ ▷ Edges features

`// encode queries from node state`
$Q_q, Q_p \leftarrow f_{nqm}(X_n) \in \mathbb{R}^{N\times N_h\times N_k} \times \mathbb{R}^{N\times N_h\times N_k}$ ▷ Encoded queries

`// encode keys from edges state`
$K_q \leftarrow f_{eqkm}(X_e) \in \mathbb{R}^{N\times n\times N_k}$ ▷ Scalar keys
$K_p \leftarrow f_{epkm}(X_e) \in \mathbb{R}^{N\times 3n\times N_k}$ ▷ Vector keys

`// encode values from edges state`
$V_q, V_p \leftarrow f_{evm}(X_e) \in \mathbb{R}^{N\times n\times S} \times \mathbb{R}^{N\times n\times S}$ ▷ Edges encoded values
$\vec{X}_g \leftarrow \text{concat}(V_p \odot \vec{r}_{nn}, \vec{p}, \vec{p}_{nn}) \in \mathbb{R}^{N\times 3n\times S\times 3}$ ▷ Geometric features

`// scaled dot-product attention and projection`
$q_h \leftarrow f_{qpm}(\text{softmax}(\frac{Q_q K_q^T}{\sqrt{N_k}})V_q) \in \mathbb{R}^{N\times S}$ ▷ Scalar hidden state
$\vec{p}_h \leftarrow W_{ppm}\ \text{softmax}(\frac{Q_p K_p^T}{\sqrt{N_k}})\vec{X}_g \in \mathbb{R}^{N\times S\times 3}$ ▷ Vectorial hidden state

`// update state with residual`
$q' \leftarrow q + q_h$
$\vec{p}' \leftarrow \vec{p} + \vec{p}_h$

Supplementary Algorithm 1 | PeSTo geometric transformer. Each geometric transformer is composed of 5 neural networks of 3 layers with an exponential linear unit (ELU) activation function. The characteristic dimensions are the number of atoms (*N*), the state size (*S*), the number of nearest neighbors (*nn*), the dimension of the embedding for the keys (N_k) and the number of attention heads (N_h). The neural networks haven a flat architecture with hidden layers width equal to the input and output state size (*S*). The multi-layers perceptrons (MLP) are the node query model (f_{nqm}), encoding scalar key model (f_{eqkm}), encoding vector key model (f_{epkm}), encoding value model (f_{evm}), and scalar state projection model (f_{qpm}). The vectorial hidden state is projected over the attention heads with a weighted sum (W_{ppm}) to preserve the rotation equivariance of the operation. The output vector state belongs to the span of the geometry and vector states.

Algorithm 2: Geometric pooling

Input: Center node features: $q \in \mathbb{R}^{N \times S}, p \in \mathbb{R}^{N \times S \times 3}$
Atoms to residue map: $M \in \{0, 1\}^{N \times N_r}$
Output: States of residue nodes: $q_r, \vec{p}_r$

```
// pack node features and define atoms to residue attention filter
```

$X_n \leftarrow concat(q, \|\vec{p}\|) \in \mathbb{R}^{N \times 2S}$ ▷ Node features

$F \leftarrow \frac{1 - M + \epsilon}{M - \epsilon} \in \mathbb{R}^{N \times N_r}$ ▷ Attention filter

```
// compute multi-heads residue-localized self-attention masks
```

$Z_q, Z_p \leftarrow f_{sam}(X_n) \in \mathbb{R}^{N \times N_h} \times \mathbb{R}^{N \times N_h}$ ▷ Multi-heads self-attention amplitudes

$A_q \leftarrow \text{softmax}(Z_q + F) \in \mathbb{R}^{N \times N_r \times N_h}$ ▷ Scalar state attention

$A_p \leftarrow \text{softmax}(Z_p + F) \in \mathbb{R}^{N \times N_r \times N_h}$ ▷ Vector state attention

```
// apply attention and projection to the input state
```

$q_r \leftarrow f_{qrpm}(A_q q) \in \mathbb{R}^{N_r \times S}$

$\vec{p}_r \leftarrow W_{prpm} A_p \vec{p} \in \mathbb{R}^{N_r \times S \times 3}$

Supplementary Algorithm 2 | PeSTo geometric pooling. Each geometric transformer is composed of 2 neural networks of 3 layers with an exponential linear unit (ELU) activation function. The characteristic dimensions are the number of atoms (*N*), the number of residues (N_r), the state size (*S*), the dimension of the embedding for the keys (N_k) and the number of attention heads (N_h). The neural networks have a flat architecture with hidden layers width equal to the input and output state size (*S*). The multi-layers perceptrons (MLP) are the self-attention model (f_{sam}) and residue scalar state projection model (f_{qpm}). The vectorial hidden state is projected with a weighted sum (W_{prpm}) to preserve the rotation equivariance of the operation.

Supplementary Table 1 | Detailed comparison of PeSTo with ScanNet. We compared our method against ScanNet on the set of structures shared both by the test set of ScanNet and our own test set (i.e., 417 structures in common). A detailed comparison is also performed for all the four different testing sets with different cutoff criteria (e.g. sequence identity of at least 70% (1), Homology (2), Topology (3) and None (4)) as defined by the ScanNet[15]. The metrics provided are the median receiver operating characteristics (ROC) area under the curve (AUC), the median precision-recall (PR) area under the curve (AUC) and the median Matthews correlation coefficient (MCC).

		ROC AUC			PR AUC			MCC		
subset	subset size	PeSTo	ScanNet-noMSA	ScanNet	PeSTo	ScanNet-noMSA	ScanNet	PeSTo	ScanNet-noMSA	ScanNet
Test (All)	417	**0.929**	0.869	0.897	**0.797**	0.667	0.72	**0.636**	0.434	0.51
Test (70\%)	98	**0.912**	0.854	0.893	**0.794**	0.671	0.782	**0.593**	0.402	0.56
Test (Homology)	133	**0.906**	0.854	0.887	**0.753**	0.604	0.691	**0.576**	0.418	0.494
Test (Topology)	87	**0.971**	0.916	0.921	**0.9**	0.748	0.796	**0.769**	0.522	0.579
Test (None)	99	0.842	0.841	**0.85**	**0.587**	0.542	0.512	**0.461**	0.338	0.363

Supplementary Table 2 | Benchmark of PeSTo against AlphaFold-multimer. Benchmark performed on 23 dimers (46 interfaces) selected from the structures within the validation set of PeSTo and AlphaFold. The accuracy, precision, Matthews correlation coefficient (MCC), receiver operating characteristic (ROC) and precision-recall (PR) area under the curve (AUC) are computed on the predictions from PeSTo and the average interfaces over the 5 AlphaFold-multimer predicted models.

	Accuracy	Precision	MCC	ROC AUC	PR AUC
**PeSTo**	0.89	0.69	0.67	0.94	0.84
**AlphaFold-multimer**	0.93	0.77	0.77	0.94	0.88

Supplementary Table 3 | Detailed benchmark of PeSTo for the molecular dynamics (MD) dataset. Benchmark of protein-protein interfaces prediction for the bound, unbound and MD sampled conformations. The benchmark dataset contains on 20 complexes (40 subunits in the bound state) and 40 subunits alone (unbound state). We sample conformations from 1µs-long MD simulation for each of the 80 subunits alone. The metrics provided includes the receiver operating characteristics (ROC) area under the curve (AUC), the precision-recall (PR) area under the curve (AUC) and the Matthews correlation coefficient (MCC).

	Bound	Unboud	Sampled
Accuracy	0.87	0.85	0.83
Precision	0.68	0.56	0.43
Negative Predictive Value	0.88	0.87	0.88
True Positive Rate	0.06	0.08	0.21
True Negative Rate	0.99	0.98	0.94
MCC	0.39	0.26	0.22
ROC AUC	0.85	0.82	0.79
PR AUC	0.52	0.45	0.42
F1 score	0.11	0.12	0.2
Prevalence	0.13	0.13	0.13

Supplementary Table 4 | Detailed benchmark of PeSTo. Benchmark of protein-protein, -nucleic acid, -ion, -ligand and -lipid interfaces prediction. The benchmark dataset contains on 512 structures randomly sampled from the testing dataset per interface type for the protein, ion and ligand interfaces predictions, 391 and 161 structures for the nucleic acid and lipid interfaces predictions, respectively. The metrics provided includes the receiver operating characteristics (ROC) area under the curve (AUC), the precision-recall (PR) area under the curve (AUC) and the Matthews correlation coefficient (MCC).

	Protein	Nucleic Acid	Ion	Ligand	Lipid
Accuracy	0.89	0.9	0.93	0.89	0.69
Precision	0.74	0.46	0.4	0.36	0.12
Negative Predictive Value	0.92	0.96	0.98	0.97	0.98
True Positive Rate	0.65	0.63	0.64	0.6	0.72
True Negative Rate	0.94	0.93	0.95	0.92	0.69
MCC	0.62	0.48	0.47	0.41	0.2
ROC AUC	0.91	0.89	0.87	0.86	0.77
PR AUC	0.76	0.56	0.6	0.55	0.13
F1 score	0.69	0.53	0.49	0.45	0.21
Prevalence	0.19	0.09	0.05	0.07	0.06

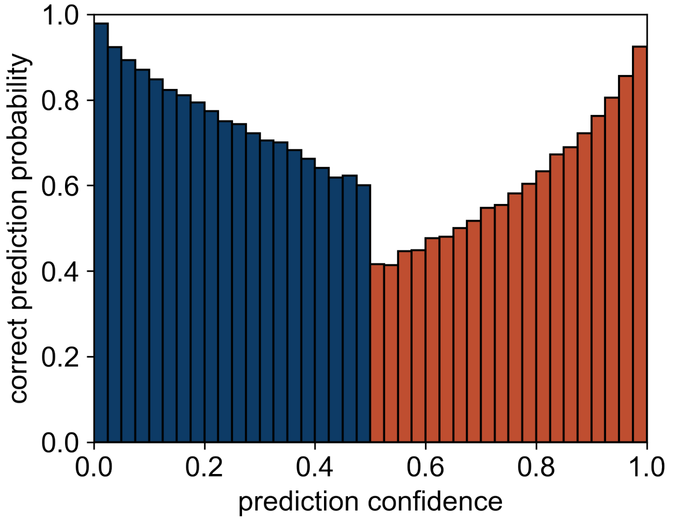

Supplementary Figure 1 | Prediction quality estimation. Estimated correlation between protein-protein interface prediction confidence and prediction quality. Evaluated on 8192 structures randomly sampled from the testing dataset.

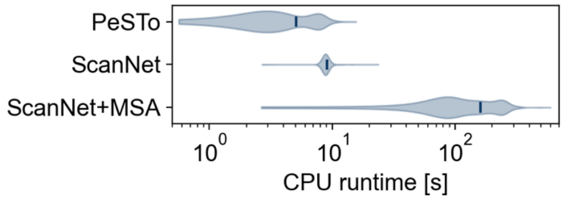

Supplementary Figure 2 | Runtime comparison of PeSTo with ScanNet. We compare PeSTo to ScanNet with and without multiple sequence alignment (MSA) on CPU (Intel i9-9900K) using 417 structures from the ScanNet benchmark dataset. We show the CPU runtime the distribution (shaded) and mean of the distribution (line) of each method.

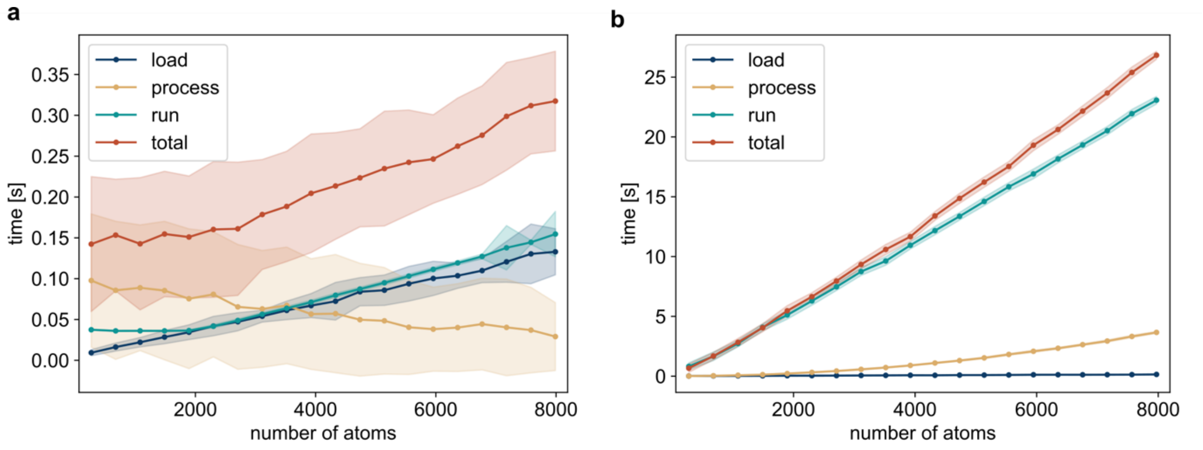

Supplementary Figure 3 | Profiling of the run time of PeSTo as a function of the size of the structure. Run time evaluated (a) on GPU (NVIDIA RTX 2080 Ti) and (b) on CPU only (Intel i9-9900K). For structures of around 100 kDa (8000 atoms), the average total runtime is 300 ms with 130 ms to parse the file, 30ms to process the structure and 140 ms to run the inference on a high-end GPU. Data are presented as the mean ± standard deviation using (a) n=194 and (b) n=19 randomly sampled structures from our test set per range of number of atoms.

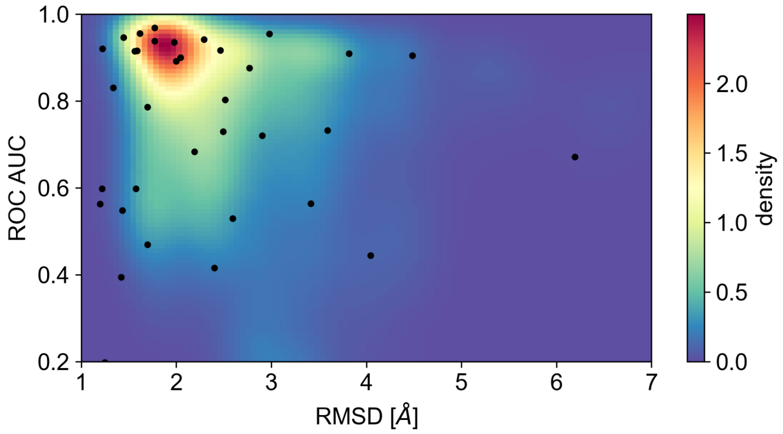

Supplementary Figure 4 | ROC AUC as a function of RMSD for different conformations for the 80 simulated subunits from the PPDB5 dataset. The RMSD is computed from the bound conformation of the subunits in the reference complex. Starting conformations are indicated with a black dot.

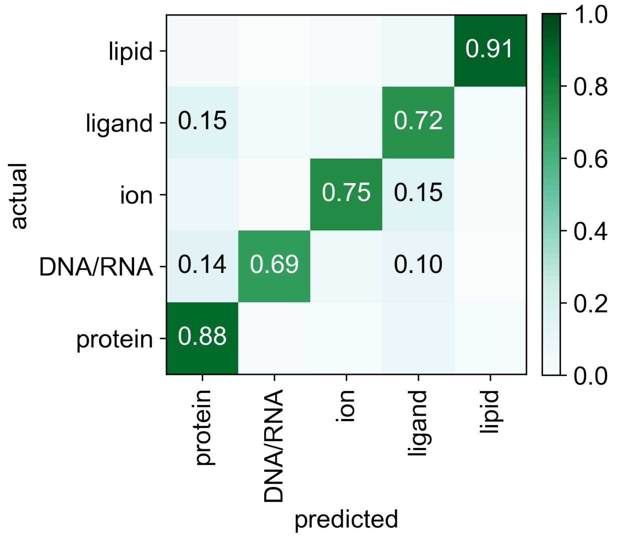

Supplementary Figure 5 | Confusion matrix between actual and predicted interfaces at the residue level. Each interface type is randomly sampled equally at ~3600 residues per class. The confusion matrix is normalized per actual interface. We observed nucleoside triphosphates (ATP, GTP) and diphosphates (ADP, GDP) pockets misclassified as nucleic acid binding regions, a reasonable confusion given the chemical similarity of all these molecules.

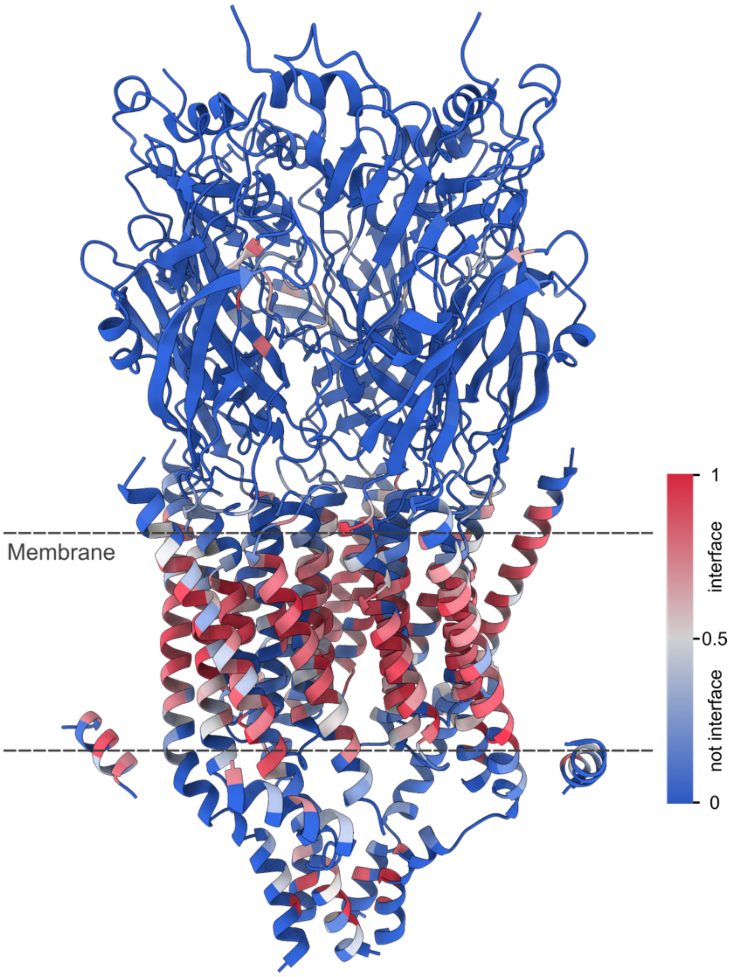

Supplementary Figure 6 | Example of lipid interface prediction for transmembrane protein. Homopentameric 5-HT 3A serotonin receptor (PDB ID: 6Y5B).

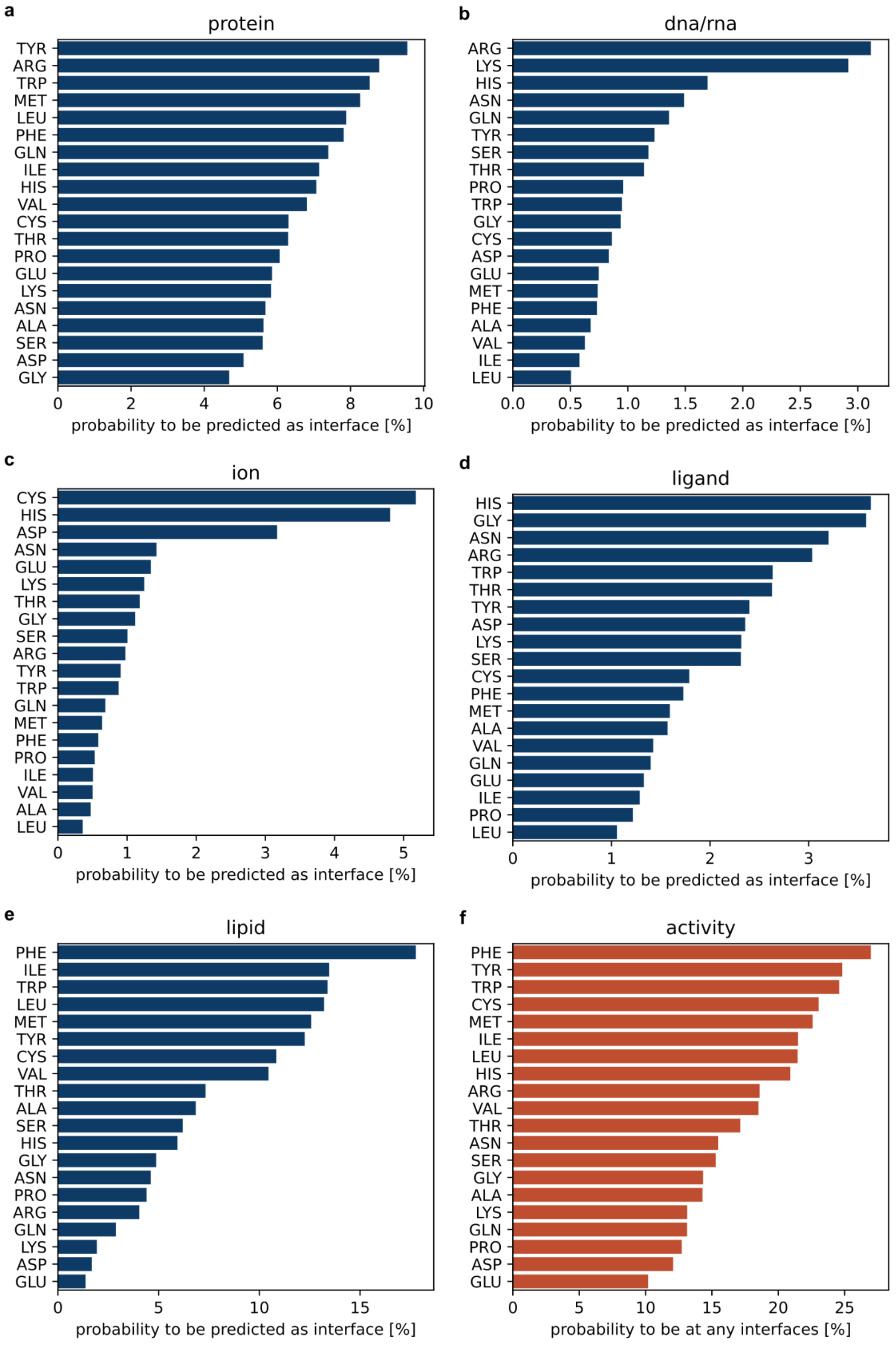

Supplementary Figure 7 | Probability of residues to be at a predicted interface. (**a-e**) Probability of different amino acids to be at a protein-protein, -nucleic acid, -ion, -ligand or -lipid predicted interface. (**f**) Probability of different amino acids to be at any interacting interfaces.

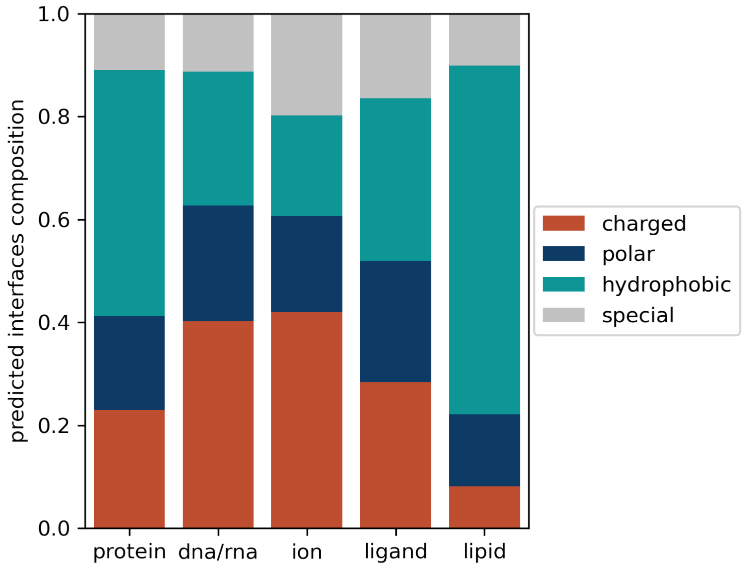

Supplementary Figure 8 | Predicted interface composition. Charged (ARG, HIS, LYS, ASP, GLU), polar (SER, THR, ASN, GLN), hydrophobic (ALA, VAL, ILE, LEU, MET, PHE, TYR), and special (CYS, GLY, PRO) residue composition for the different predicted interface types.

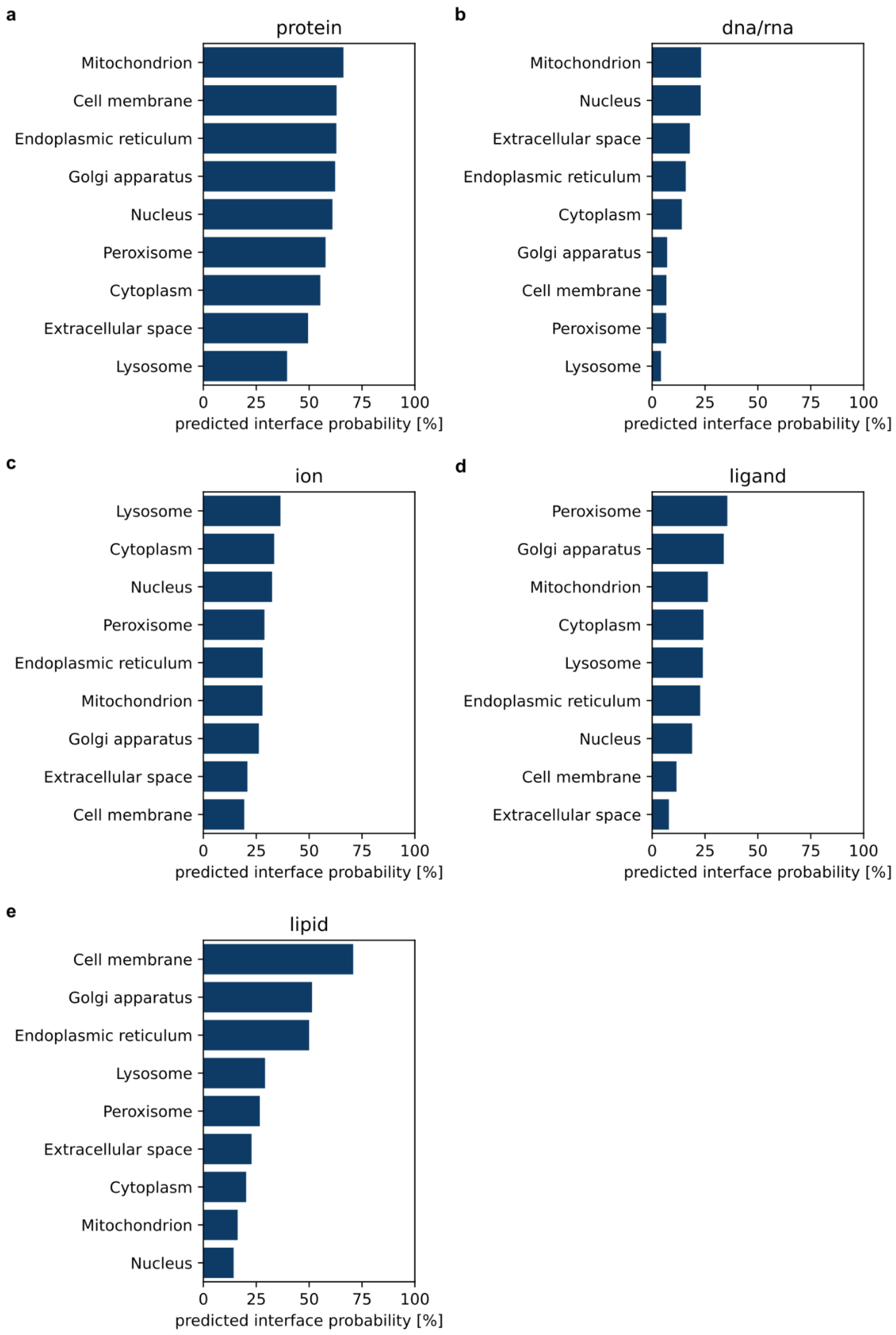

Supplementary Figure 9 | Subcellular localization. Probability of a protein within a specified subcellular localization to have an interface with (**a**) protein, (**b**) nucleic acid, (**c**) ion, (**d**) ligand, (**e**) lipid.

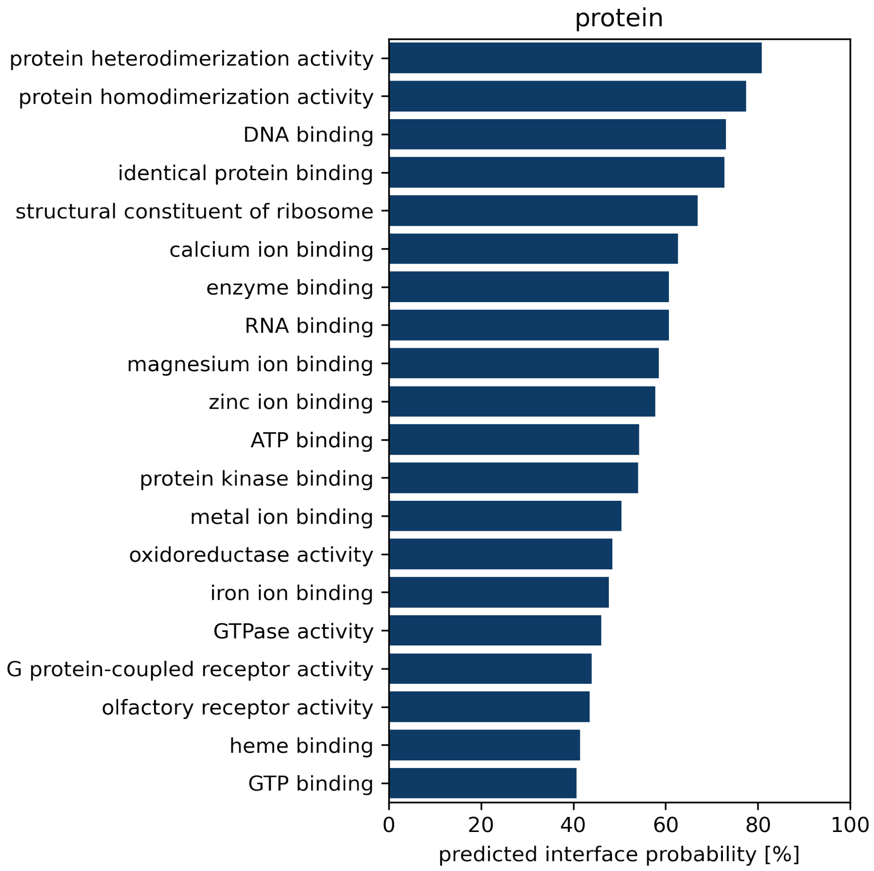

Supplementary Figure 10 | GO molecular function for protein-protein interfaces. Probability of protein with the specified molecular function to have a protein-protein interface (Minimum sampling of 200 examples per GO term).

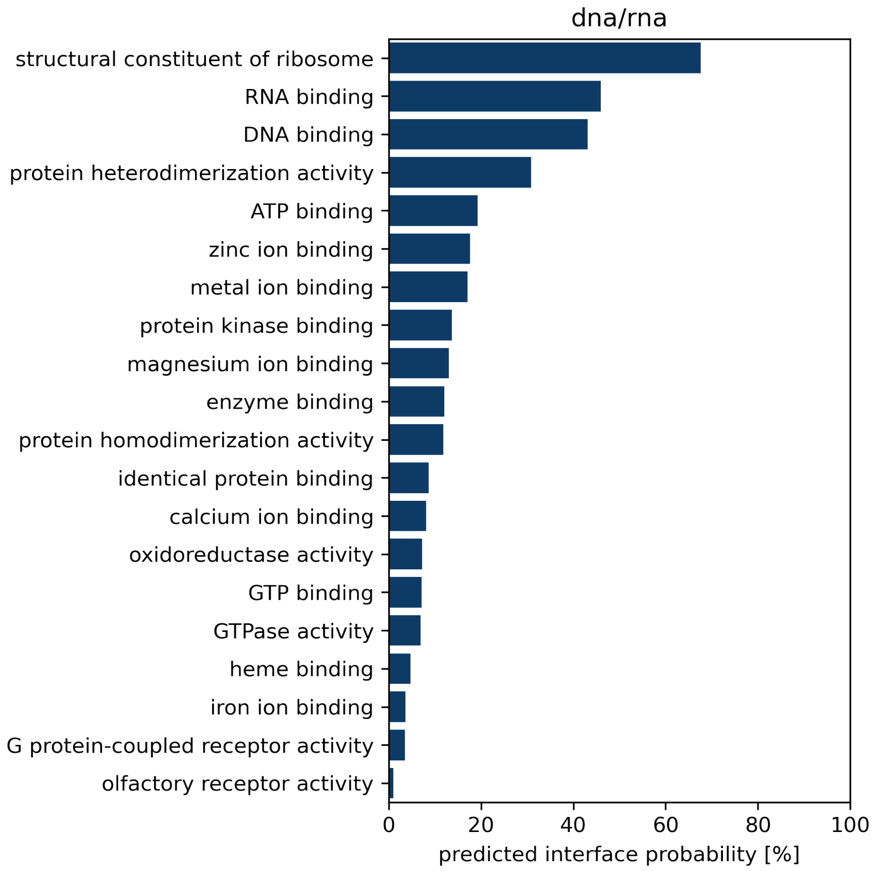

Supplementary Figure 11 | GO molecular function for protein-nucleic acid interfaces. Probability of protein with the specified molecular function to have a protein-nucleic acid interface (Minimum sampling of 200 examples per GO term).

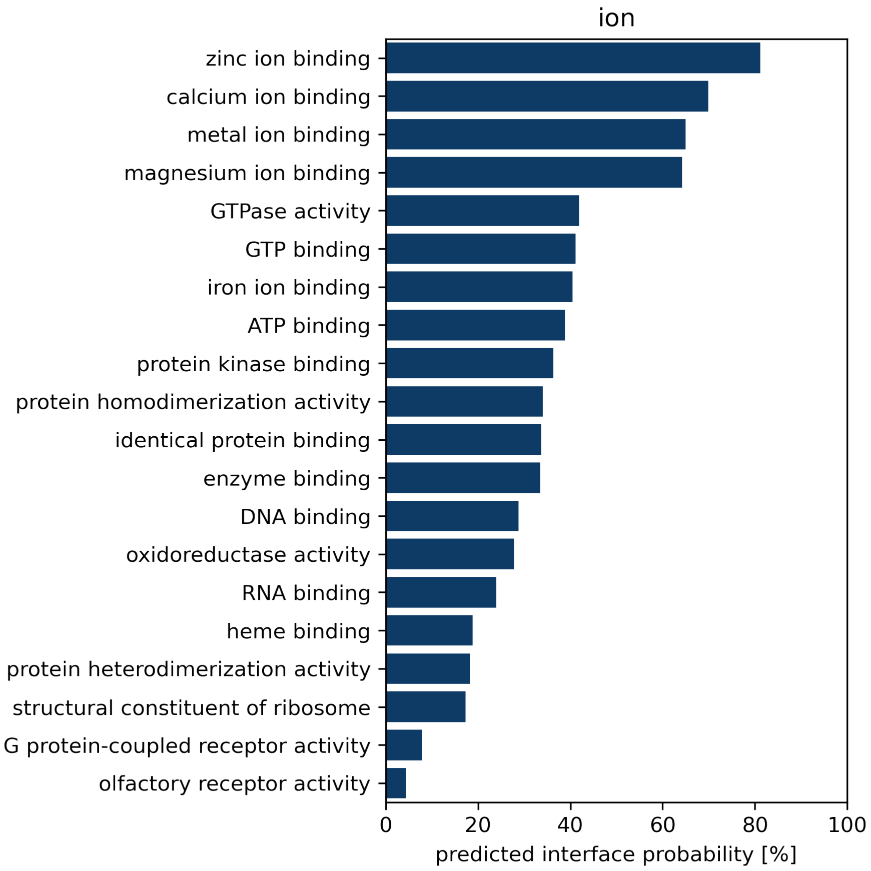

Supplementary Figure 12 | GO molecular function for protein-ion interfaces. Probability of protein with the specified molecular function to have a protein-ion interface (Minimum sampling of 200 examples per GO term).

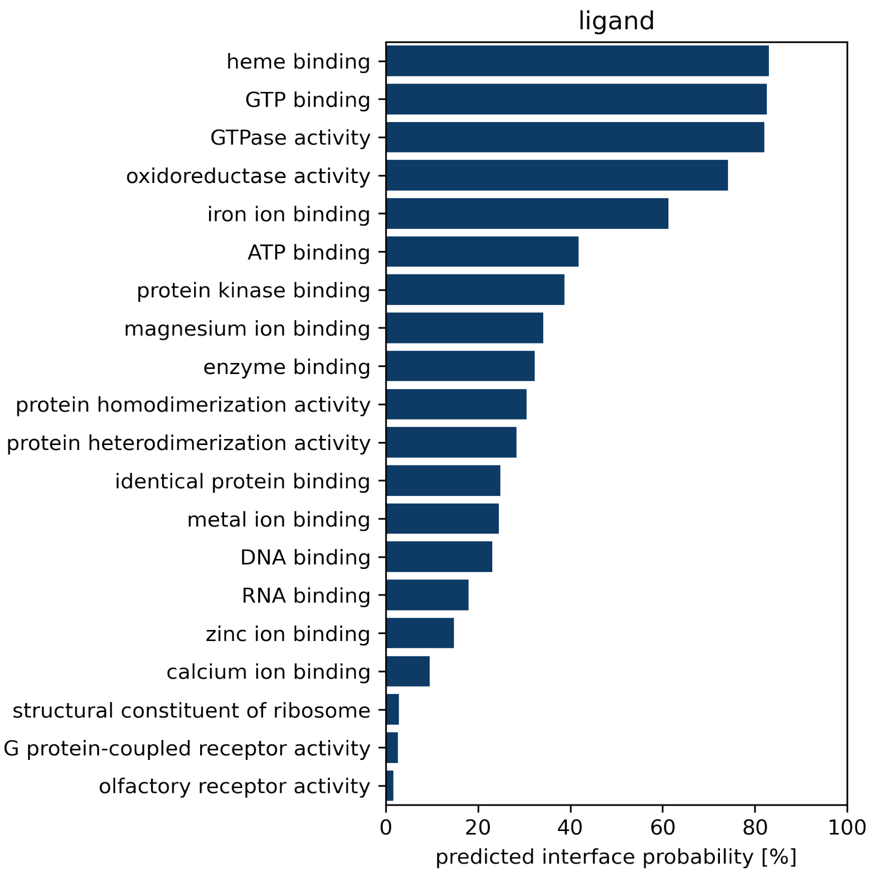

Supplementary Figure 13 | GO molecular function for protein-ligand interfaces. Probability of protein with the specified molecular function to have a protein-ligand interface (Minimum sampling of 200 examples per GO term).

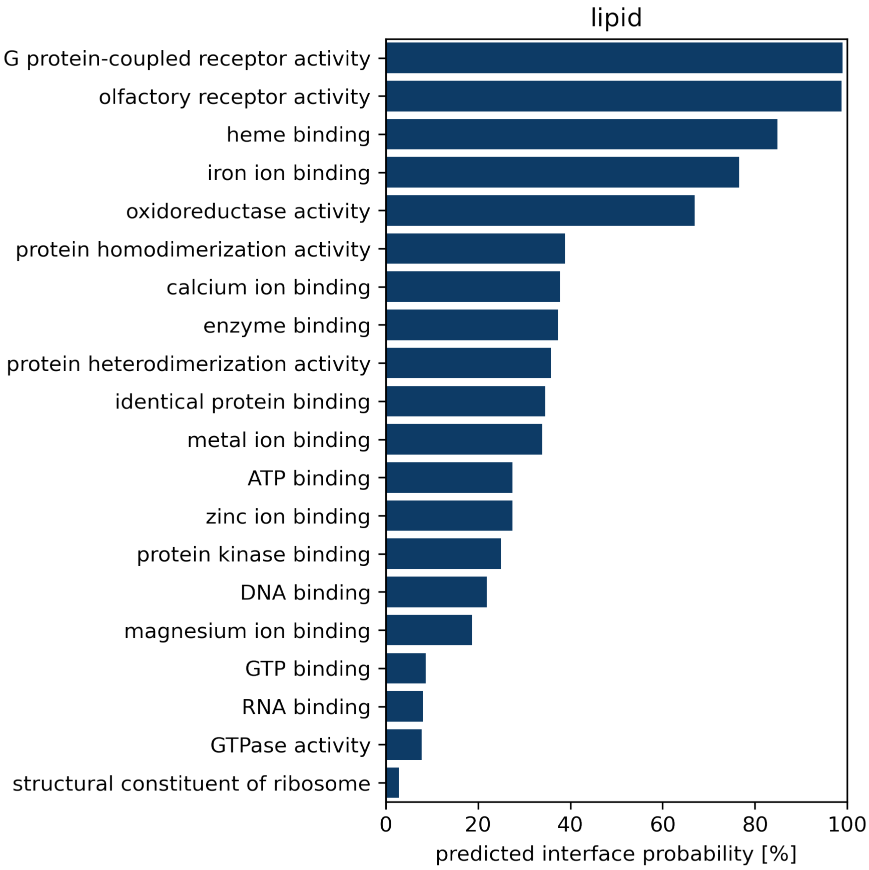

Supplementary Figure 14 | GO molecular function for protein-lipid interfaces. Probability of protein with the specified molecular function to have a protein-lipid interface (Minimum sampling of 200 examples per GO term).

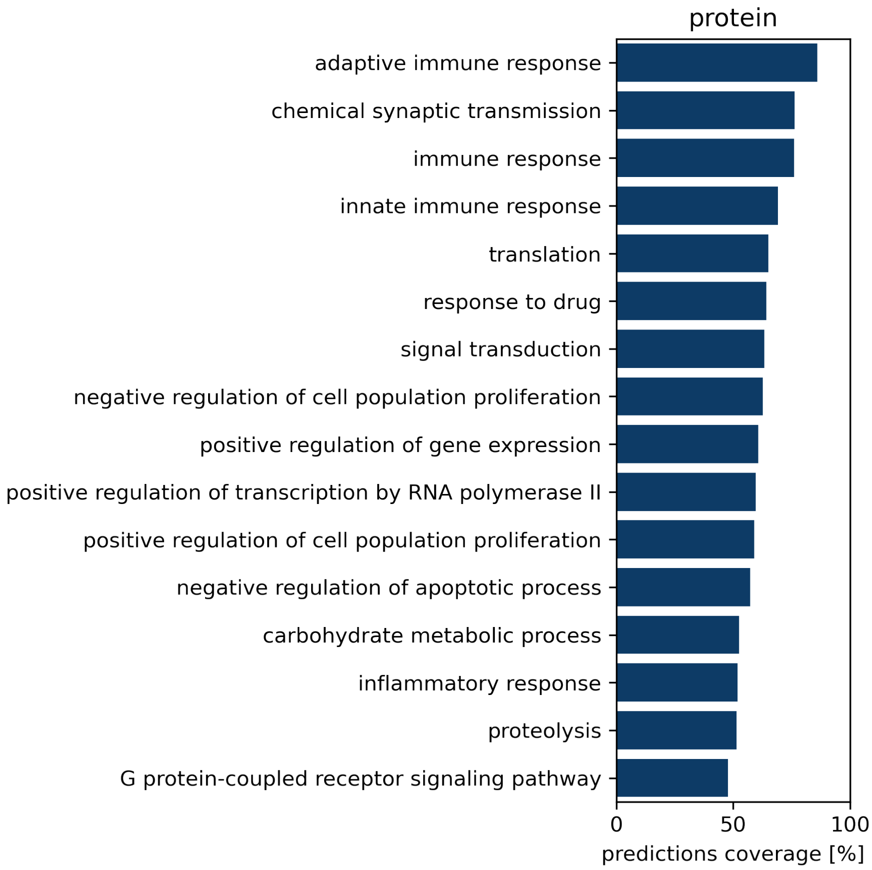

Supplementary Figure 15 | GO biological process for protein-protein interfaces. Probability of protein with the specified biological process to have a protein-protein interface (Minimum sampling of 200 examples per GO term).

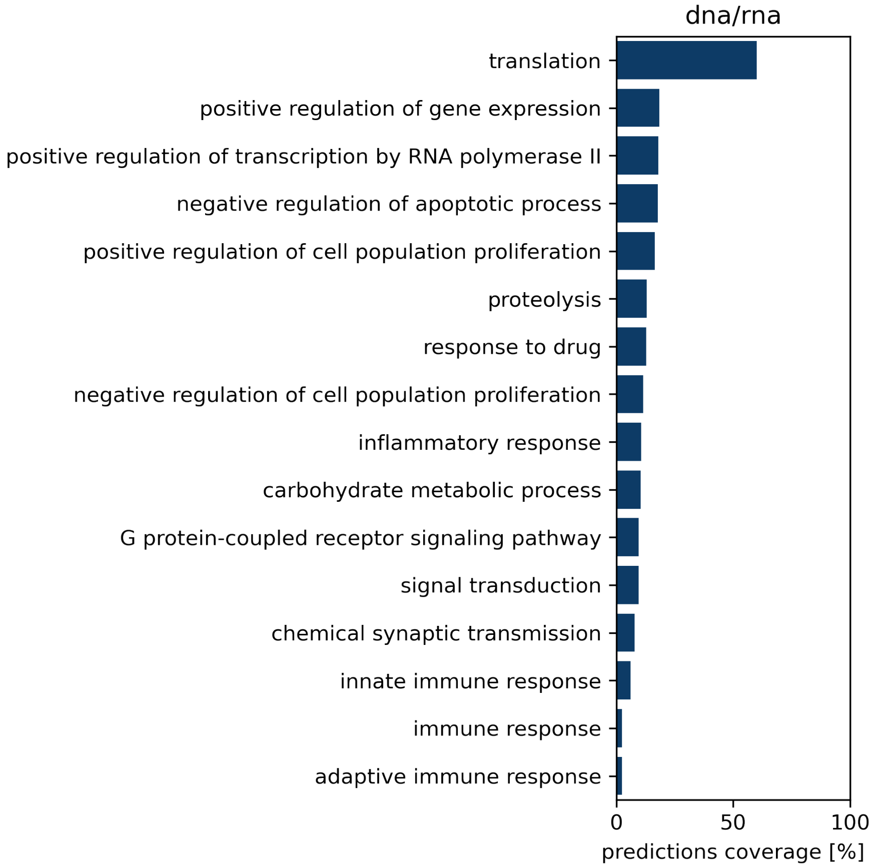

Supplementary Figure 16 | GO biological process for protein-nucleic acid interfaces. Probability of protein with the specified biological process to have a protein-nucleic acid interface (Minimum sampling of 200 examples per GO term).

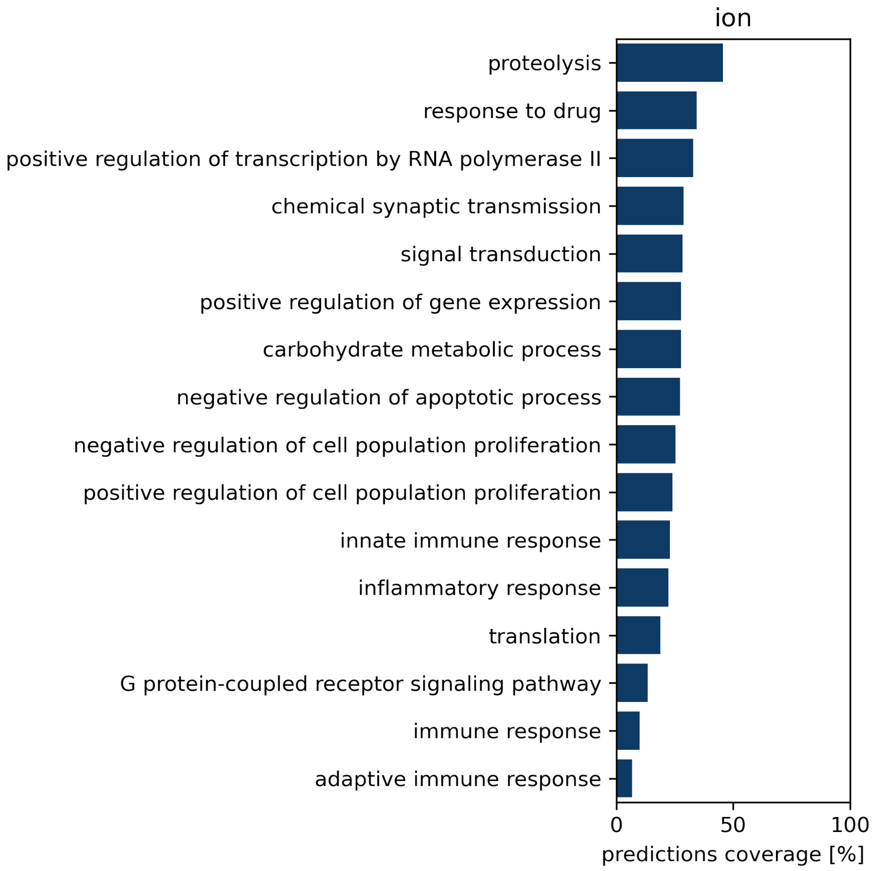

Supplementary Figure 17 | GO biological process for protein-ion interfaces. Probability of protein with the specified biological process to have a protein-ion interface (Minimum sampling of 200 examples per GO term).

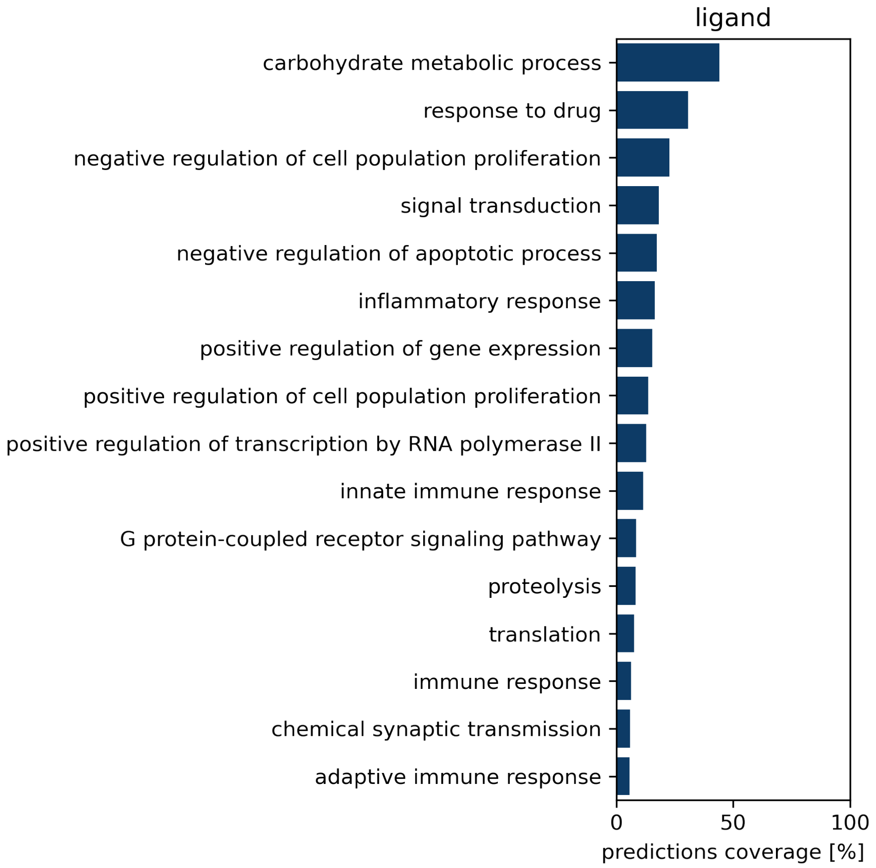

Supplementary Figure 18 | GO biological process for protein-ligand interfaces. Probability of protein with the specified biological process to have a protein-ligand interface (Minimum sampling of 200 examples per GO term).

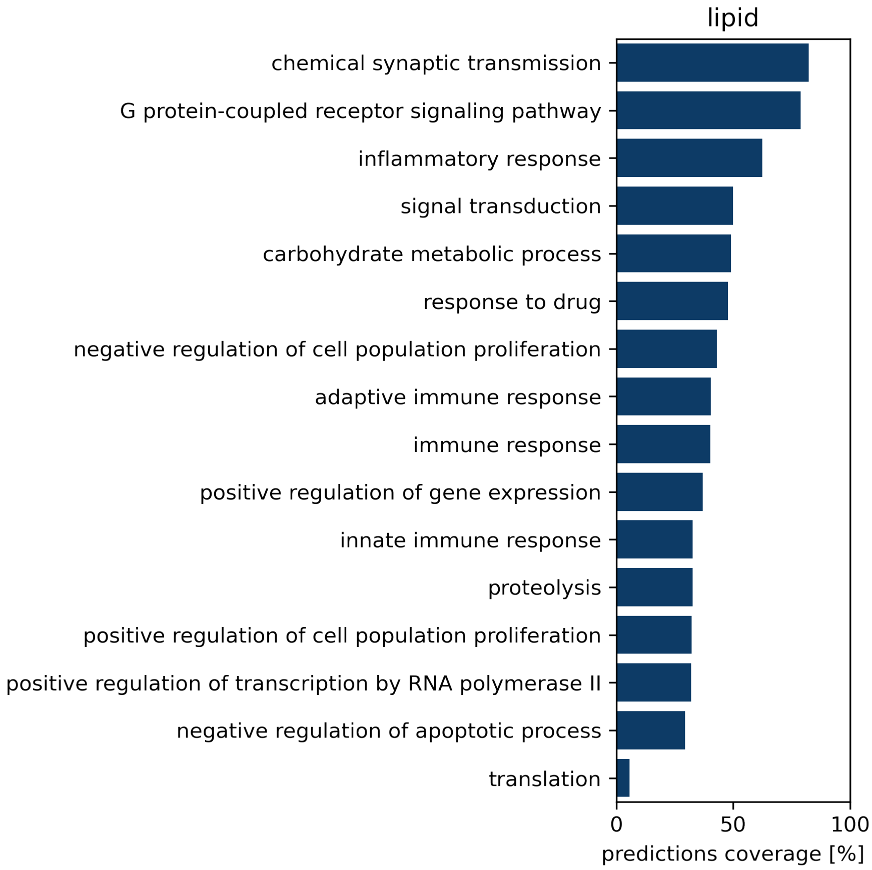

Supplementary Figure 19 | GO biological process for protein-lipid interfaces. Probability of protein with the specified biological process to have a protein-lipid interface (Minimum sampling of 200 examples per GO term).

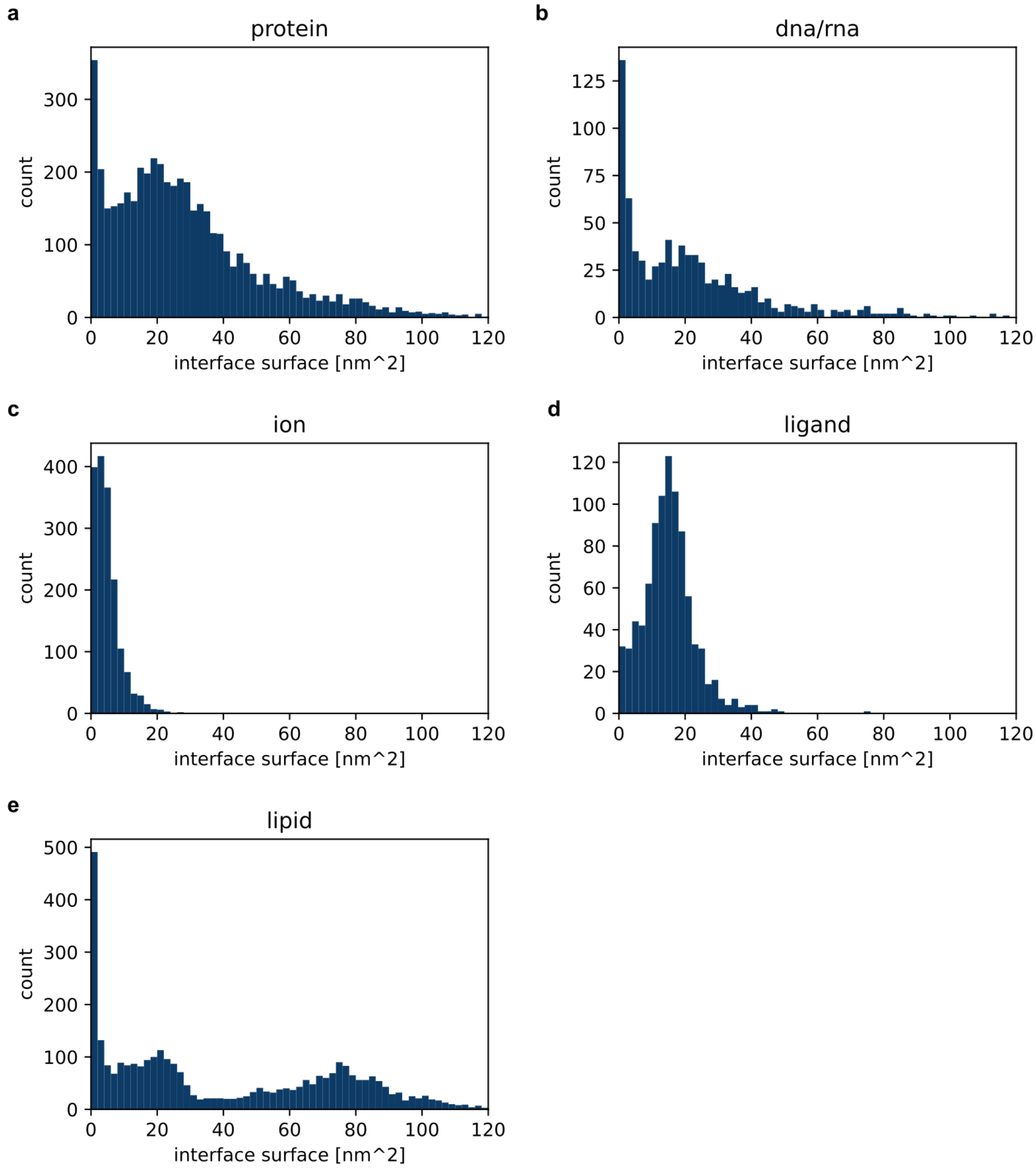

Supplementary Figure 20 | Solvent accessible surface area. Solvent accessible surface area distribution of predicted interfaces for protein (**a**) -protein (32±22 nm^2), (**b**) -nucleic acid (29±23 nm^2), (**c**) -ion (7±4 nm^2), (**d**) -ligand (16±7 nm^2), and (**e**) -lipid (17±9 nm^2 & 75±19 nm^2).

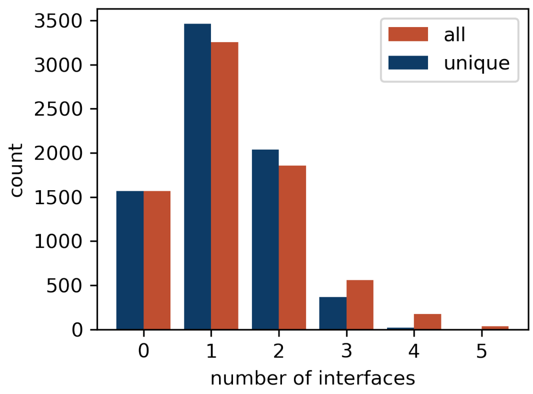

Supplementary Figure 21 | Number of interfaces. Distribution of the number of (different) interfaces per subunit. The total number of disjoint interfaces is shown as “all” interfaces count. The number of different type of interfaces (i.e. protein-protein, -nucleic acid, -ion, -ligand and -lipid interfaces) is indicated as the “unique” interfaces count.

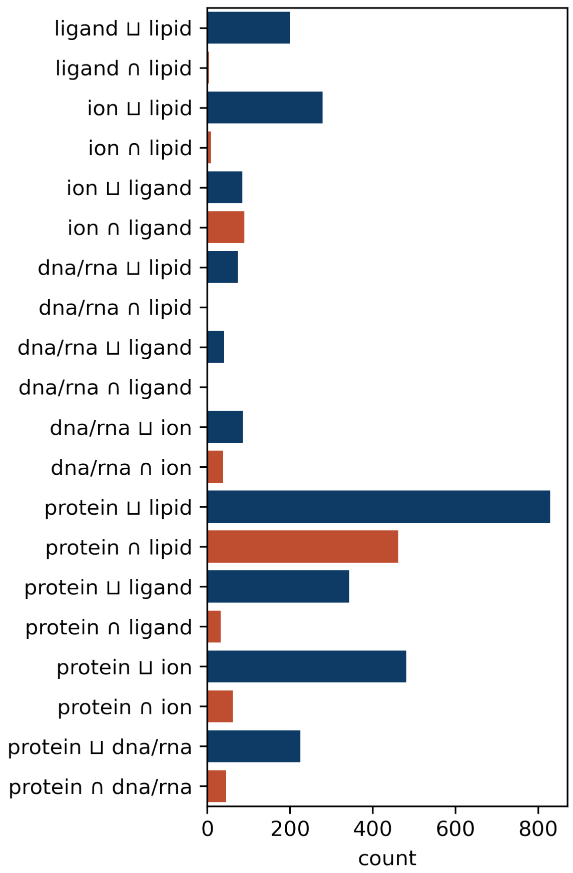

Supplementary Figure 22 | Intersecting and disjoint interfaces. Number of interfaces that contained two types of interfaces either overlapped (∩) or not (⊔).

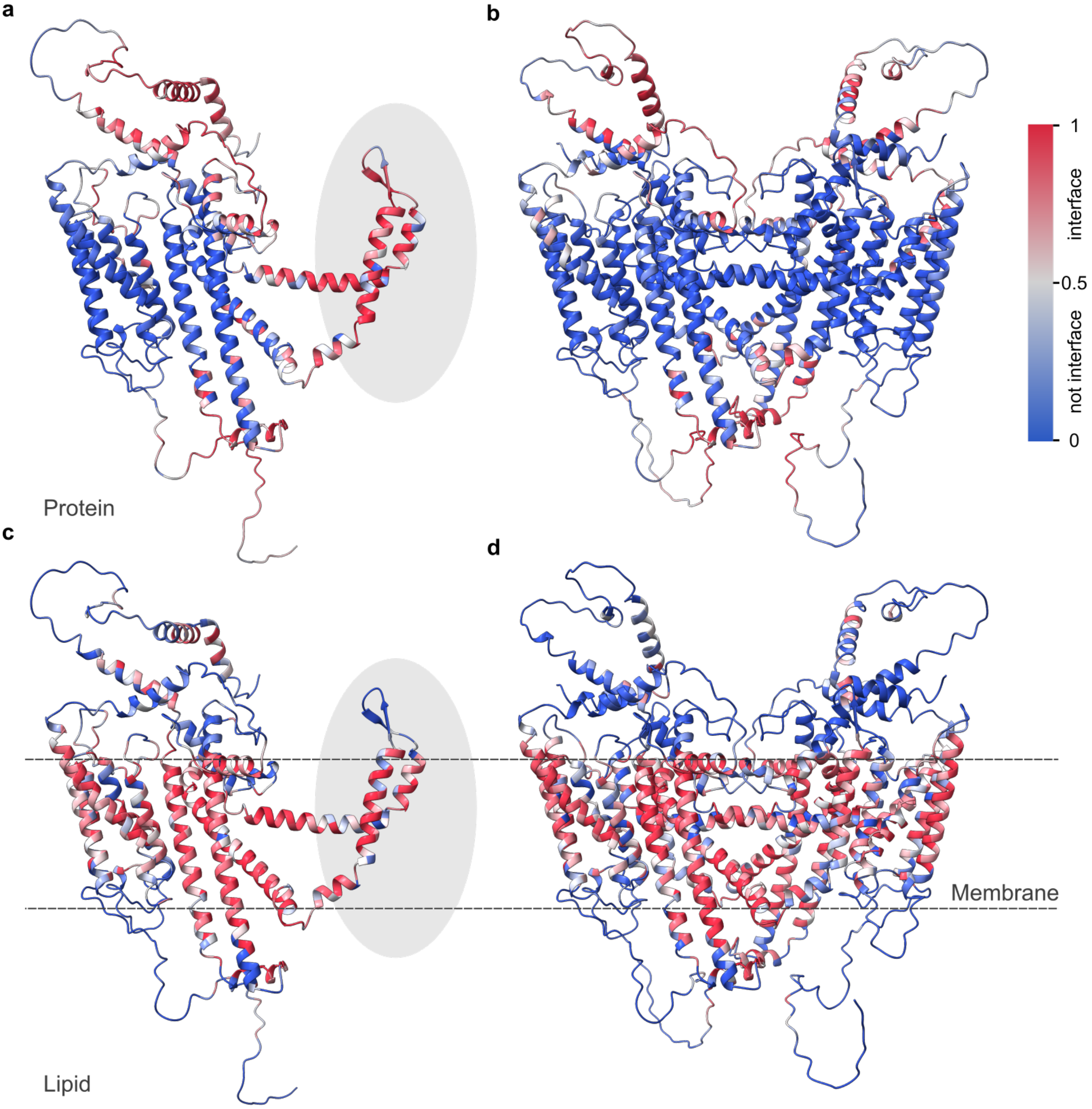

Supplementary Figure 23 | Details for STRA6 example (UniProt Q9BX79). PeSTo predicted interfaces for (**a**,**b**) protein-protein and (**c**,**d**) protein-lipid interactions with estimated membrane location. (**a**,**c**) AlphaFold predicted monomers with highlighted protein-protein and protein-lipid interfaces overlap. (**b**,**d**) AlphaFold-multimer predicted dimers.

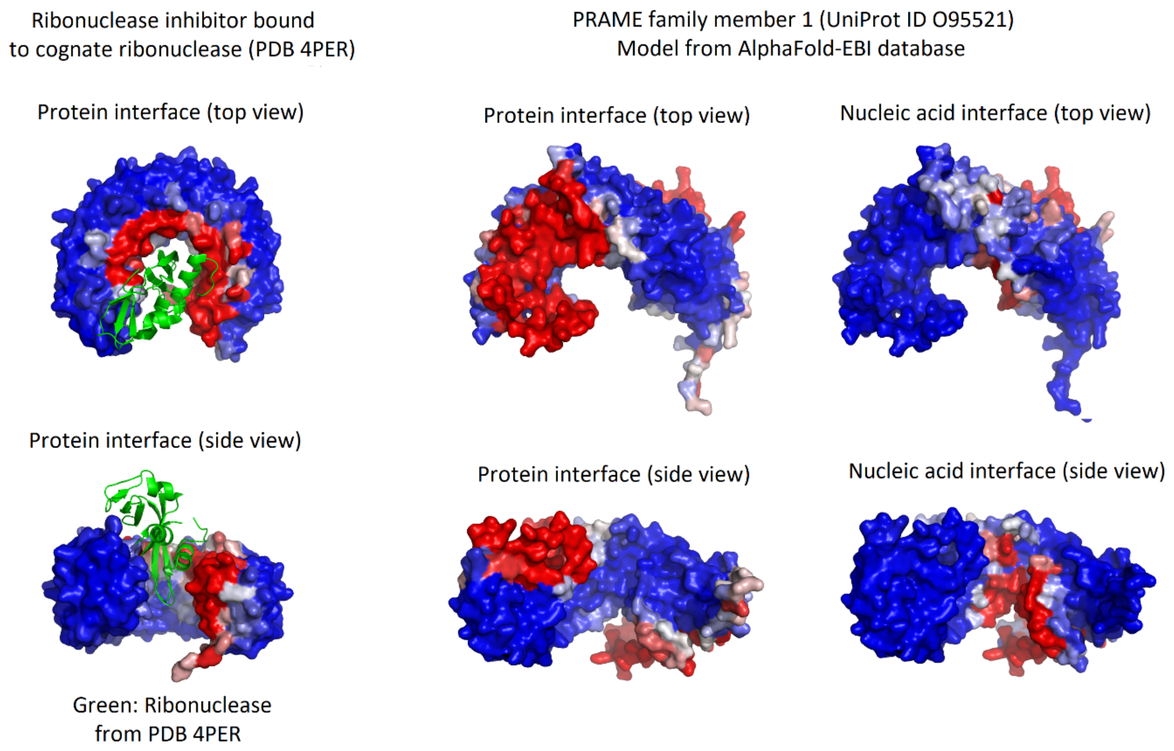

Supplementary Figure 24 | Details for PRAMEfm1 example (UniProt O95521). Top and side views of surface representations highlighting PeSTo-predicted interfaces with proteins and nucleic acids for a high-confidence AlphaFold model of PRAMEfm1 (right) and for a structurally related ribonuclease inhibitor of structure solved in complex with its cognate ribonuclease (left, from PDB 4PER).
