# Supplementary material for: PeSTo: parameter-free geometric deep learning for accurate prediction of protein binding interfaces
Source: Nat Commun. 2023 Apr 18;14:2175. doi: 10.1038/s41467-023-37701-8 (PMC10113261; doi:10.1038/s41467-023-37701-8)
Supplement: Supplementary file 3 — Description of Additional Supplementary Files [file 41467_2023_37701_MOESM3_ESM.pdf]

Description of Additional Supplementary Files

File Name: Supplementary Data 1
Description: The curated data concerning the human interfaceome.
