# Supplementary material for: PeSTo: parameter-free geometric deep learning for accurate prediction of protein binding interfaces
Source: Nat Commun. 2023 Apr 18;14:2175. doi: 10.1038/s41467-023-37701-8 (PMC10113261; doi:10.1038/s41467-023-37701-8)
Supplement: Supplementary file 5 — Reporting Summary [file 41467_2023_37701_MOESM5_ESM.pdf]

nature research

Corresponding author(s): Matteo Dal Peraro

Last updated by author(s): Mar 20, 2023

Reporting Summary

Nature Research wishes to improve the reproducibility of the work that we publish. This form provides structure for consistency and transparency in reporting. For further information on Nature Research policies, see our Editorial Policies and the Editorial Policy Checklist.

Statistics

For all statistical analyses, confirm that the following items are present in the figure legend, table legend, main text, or Methods section.

n/a	Confirmed	
☐	☒	The exact sample size (*n*) for each experimental group/condition, given as a discrete number and unit of measurement
☒	☐	A statement on whether measurements were taken from distinct samples or whether the same sample was measured repeatedly
☒	☐	The statistical test(s) used AND whether they are one- or two-sided<br>*Only common tests should be described solely by name; describe more complex techniques in the Methods section.*
☒	☐	A description of all covariates tested
☒	☐	A description of any assumptions or corrections, such as tests of normality and adjustment for multiple comparisons
☐	☒	A full description of the statistical parameters including central tendency (e.g. means) or other basic estimates (e.g. regression coefficient) AND variation (e.g. standard deviation) or associated estimates of uncertainty (e.g. confidence intervals)
☒	☐	For null hypothesis testing, the test statistic (e.g. F, t, r) with confidence intervals, effect sizes, degrees of freedom and P value noted<br>*Give P values as exact values whenever suitable.*
☒	☐	For Bayesian analysis, information on the choice of priors and Markov chain Monte Carlo settings
☒	☐	For hierarchical and complex designs, identification of the appropriate level for tests and full reporting of outcomes
☒	☐	Estimates of effect sizes (e.g. Cohen's *d*, Pearson's *r*), indicating how they were calculated

Our web collection on statistics for biologists contains articles on many of the points above.

Software and code

Policy information about availability of computer code

Data collection	All the code to reproduce the datasets and experiments are available at https://github.com/LBM-EPFL/PeSTo.<br>The main software and packages used are: PyTorch 1.10, NumPy 1.23.3, GEMMI 0.5.2, ColabFold v1.3.0, MMseqs2 13-45111, AlphaFold-multimer v2.1.2, GROMACS 2020.
Data analysis	All the code to reproduce the data analysis and figures are available at https://github.com/LBM-EPFL/PeSTo.<br>The main software and packages used are: JupyterLab v3.3.0, Matplotlib 3.5, ChimeraX 1.3, CLoNe, MDTraj 1.9.7.

For manuscripts utilizing custom algorithms or software that are central to the research but not yet described in published literature, software must be made available to editors and reviewers. We strongly encourage code deposition in a community repository (e.g. GitHub). See the Nature Research guidelines for submitting code & software for further information.

Data

Policy information about availability of data

All manuscripts must include a data availability statement. This statement should provide the following information, where applicable:

- Accession codes, unique identifiers, or web links for publicly available datasets
- A list of figures that have associated raw data
- A description of any restrictions on data availability

We provide instructions to access and reproduce the data at https://github.com/LBM-EPFL/PeSTo.
The databases and datasets used in this work are freely available online:
- RCSB PDB: https://www.rcsb.org/
- Protein-Protein Docking Benchmark 5.0: https://zlab.umassmed.edu/benchmark/
- AF-EBI: https://alphafold.ebi.ac.uk/

- UniProt: https://www.uniprot.org/
- dbSNP: https://www.ncbi.nlm.nih.gov/snp/

Field-specific reporting

Please select the one below that is the best fit for your research. If you are not sure, read the appropriate sections before making your selection.

☒ Life sciences ☐ Behavioural & social sciences ☐ Ecological, evolutionary & environmental sciences

For a reference copy of the document with all sections, see nature.com/documents/nr-reporting-summary-flat.pdf

Life sciences study design

All studies must disclose on these points even when the disclosure is negative.

Sample size	We used the largest amount of data publicly accessible.
Data exclusions	We excluded structures that only contains C_alpha coordinates. We established this exclusion criteria because our method uses atomic structures as input and cannot be applied to structures containing only C_alpha. Only a few structures in the full dataset are affected by this exclusion.
Replication	The results and analysis was repeated multiple times. We replicated the training for 4 models with small variations of the architecture of the neural network. The training of the 4 independent models were successful: the analysis shows comparable performance between all 4 models. We picked the best model to showcase our method. We provide the trained parameters of all 4 models at https://github.com/LBM-EPFL/PeSTo.
Randomization	We ensure that the set of structures are independent by clustering structures with more than 30% sequence identity together. The training, testing and validation set were randomly sampled from the set independent clusters. The structures were randomly sampled during training. We randomly sampled independent structures for validation and benchmarking.
Blinding	NA

Reporting for specific materials, systems and methods

We require information from authors about some types of materials, experimental systems and methods used in many studies. Here, indicate whether each material, system or method listed is relevant to your study. If you are not sure if a list item applies to your research, read the appropriate section before selecting a response.

Materials & experimental systems

n/a	Involved in the study
☒	☐ Antibodies
☒	☐ Eukaryotic cell lines
☒	☐ Palaeontology and archaeology
☒	☐ Animals and other organisms
☒	☐ Human research participants
☒	☐ Clinical data
☒	☐ Dual use research of concern

Methods

n/a	Involved in the study
☒	☐ ChIP-seq
☒	☐ Flow cytometry
☒	☐ MRI-based neuroimaging
